# Ciliary Proteins Repurposed by the Synaptic Ribbon: Trafficking Myristoylated Proteins at Rod Photoreceptor Synapses

**DOI:** 10.3390/ijms23137135

**Published:** 2022-06-27

**Authors:** Shweta Suiwal, Mayur Dembla, Karin Schwarz, Rashmi Katiyar, Martin Jung, Yvonne Carius, Stephan Maxeiner, Marcel A. Lauterbach, C. Roy D. Lancaster, Frank Schmitz

**Affiliations:** 1 Institute of Anatomy and Cell Biology, Department of Neuroanatomy, Medical School, Saarland University, 66421 Homburg, Germany; dotdot.mayur@gmail.com (M.D.); karin.schwarz@uks.eu (K.S.); katiyarrashmi81@gmail.com (R.K.); stephan.maxeiner@uni-saarland.de (S.M.); 2 Institute of Biochemistry and Molecular Biology, Medical School, Saarland University, 66421 Homburg, Germany; martin.jung@uks.eu; 3 Institute of Biophysics, Department of Structural Biology, Center of Human and Molecular Biology (ZHMB), Medical School, Saarland University, 66421 Homburg, Germany; yvonne.carius@structural-biology.eu (Y.C.); roy.lancaster@structural-biology.eu (C.R.D.L.); 4 Molecular Imaging, Center for Integrative Physiology and Molecular Medicine, Medical School, Saarland University, 66421 Homburg, Germany; marcel.lauterbach@uks.eu

**Keywords:** retina, photoreceptor synapse, synaptic ribbon, Nphp3, Arl3, Arl13b, immunogold electron microscopy

## Abstract

The Unc119 protein mediates transport of myristoylated proteins to the photoreceptor outer segment, a specialized primary cilium. This transport activity is regulated by the GTPase Arl3 as well as by Arl13b and Rp2 that control Arl3 activation/inactivation. Interestingly, Unc119 is also enriched in photoreceptor synapses and can bind to RIBEYE, the main component of synaptic ribbons. In the present study, we analyzed whether the known regulatory proteins, that control the Unc119-dependent myristoylated protein transport at the primary cilium, are also present at the photoreceptor synaptic ribbon complex by using high-resolution immunofluorescence and immunogold electron microscopy. We found Arl3 and Arl13b to be enriched at the synaptic ribbon whereas Rp2 was predominantly found on vesicles distributed within the entire terminal. These findings indicate that the synaptic ribbon could be involved in the discharge of Unc119-bound lipid-modified proteins. In agreement with this hypothesis, we found Nphp3 (Nephrocystin-3), a myristoylated, Unc119-dependent cargo protein enriched at the basal portion of the ribbon in close vicinity to the active zone. Mutations in Nphp3 are known to be associated with Senior–Løken Syndrome 3 (SLS3). Visual impairment and blindness in SLS3 might thus not only result from ciliary dysfunctions but also from malfunctions of the photoreceptor synapse.

## 1. Introduction

Photoreceptors are the principal light sensors of the retina and possess a characteristic morphology. Photoreceptors form an outer segment (OS) that is responsible for phototransduction. The OS is connected to the inner segment (IS) via a thin connecting cilium that controls transport of membranes and proteins into the OS as well as retrograde trafficking back to the IS [1,2]. The gating function of this primary cilium is important to achieve the strong enrichment of proteins of the phototransduction cascade in the OS and to adjust the sensitivity of phototransduction cascade by moving in/out regulatory components (e.g., α-transducin, arrestin) that modulate the efficacy of the phototransduction cascade during light and dark adaptation [3,4,5].

Many proteins that travel through the connecting cilium are lipid-modified and need to be inserted and removed into/from membranes [6,7]. Membrane insertion and removal at the connecting cilium is mediated by guanine nucleotide dissociation inhibitor (GDI)-like proteins, such as Unc119 and PrBP/δ [1,7,8,9,10,11,12,13,14,15,16,17]. Unc119 is essential, e.g., for the trafficking of the myristoylated proteins α-transducin and Nphp3 (Nephrocystin-3) at the connecting cilium [6,8,9,18]. In mammals, two separate Unc119 coding genes, *POC7A* and *POC7B*, produce two closely related proteins, Unc119a and Unc119b [6].

Interestingly, Unc119 is also enriched in photoreceptor presynaptic terminals [17,19,20,21] that form continuously active ribbon synapses [22,23]. Ribbon synapses have specialized active zones that contain synaptic ribbons as central presynaptic elements [24]. Readily releasable synaptic vesicles associate with the synaptic ribbon and are delivered to the active zone to promote continuous vesicle exocytosis [25,26,27]; for review, see [22,28]. RIBEYE is the main structural component of synaptic ribbons [29,30]. RIBEYE can bind Unc119a [20], raising the possibility that the photoreceptor ribbon synapses might establish similar transport mechanisms for lipid-modified proteins as the photoreceptor cilium does.

In the present study, we explored this possibility by analyzing proteins that are involved in Unc119-dependent trafficking of lipid-modified proteins. We focused on Arl3, a GTP-binding protein, that displaces the lipid-anchored protein cargo from Unc119 as a function of the bound nucleotide [14,31]. The nature of the bound nucleotide is regulated by the Retinitis Pigmentosa 2 (Rp2) protein, the GTPase-activating protein for Arl3 [32,33] and Arl13b, which is a GTP/GDP exchange factor that catalyzes replacement of GDP by GTP [34]. Arl3, Rp2 and Arl13b had been previously found in the photoreceptor cilium [2,6,14,35,36]. We also examined Nphp3, a myristoylated Unc119-dependent protein cargo at the photoreceptor cilium [9].

In our morphological study, we demonstrate that these proteins are present also at the photoreceptor synaptic ribbon complex. Our data propose that the synaptic ribbon complex is not only involved in synaptic vesicle trafficking, but also in the trafficking of lipid-modified proteins. Furthermore, we identify Nphp3 as a novel protein of the cytomatrix of the active zone in photoreceptor ribbon synapses that needs the synaptic ribbon for its efficient transport to the active zone.

## 2. Results

We previously showed that RIBEYE, the major component of synaptic ribbons [29,30], binds to Unc119 [20]. Unc119 serves as a shuttle protein for the transport of lipid-modified proteins in photoreceptors and its activity is regulated by GTP-binding proteins [6]. In the present study, we analyzed whether these regulatory proteins that control Unc119 function are present at photoreceptor synaptic ribbons. For our morphological analyses, we always used at least two different antibodies raised against each respective protein to confirm the respective protein localization by independent tools.

### 2.1. Arl3 Is Enriched at the Photoreceptor Synaptic Ribbon

**Figure 1:**
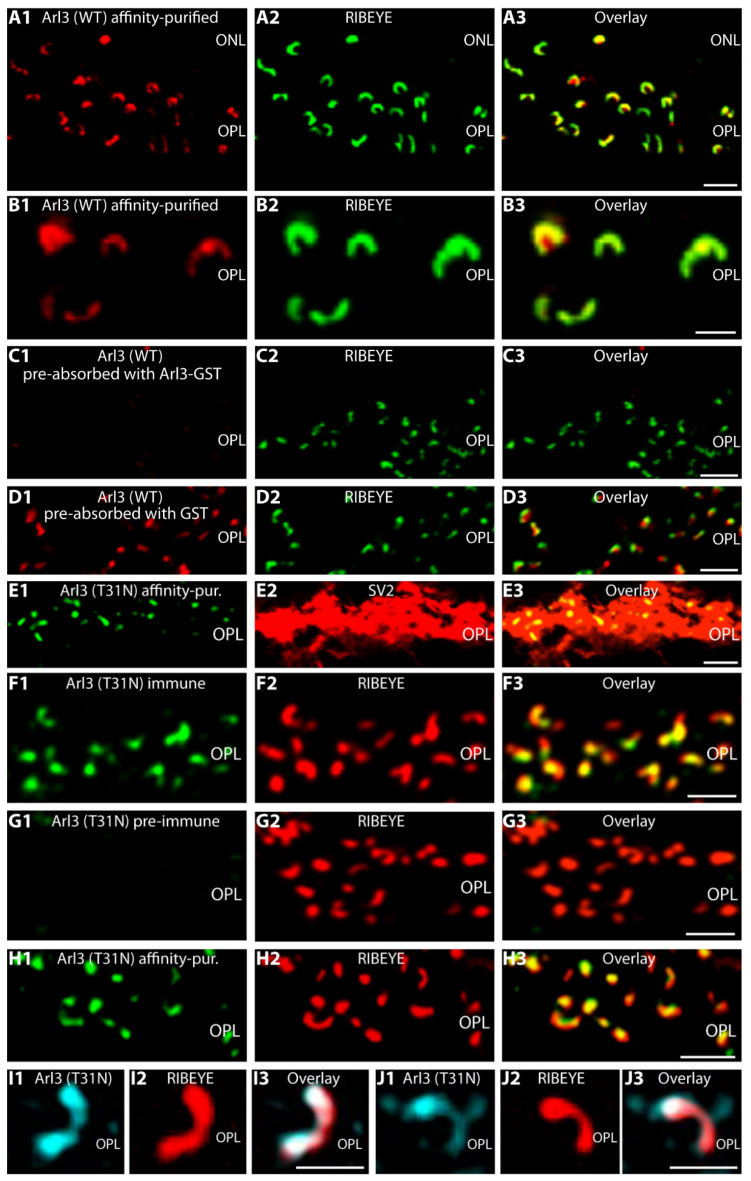
Light microscopical immunolocalization of Arl3 in photoreceptor synapses using two different Arl3 antibodies (Arl3(WT) and Arl3(T31N)) (**A**–**J**). (**A1**–**A3**,**B1**–**B3**) Cryostat sections of the bovine retina (≈10 µm thick) double-immunolabeled with affinity-purified rabbit polyclonal antibodies against Arl3 (**A1**,**B1**) and mouse monoclonal antibodies against RIBEYE(B)/CtBP2 (BD Transduction Labs) (**A2**,**B2**). Both channels are overlaid in (**A3**,**B3**). Arl3 is present in the OPL in a discrete, horseshoe-shaped manner and highly enriched at the synaptic ribbon as judged by co-localization with RIBEYE. (**C1**–**C3**,**D1**–**D3**) Preabsorption control of Arl3 antibody. Cryostat sections of the bovine retina (≈10 µm thick) immunolabeled with Arl3 antibody (Arl3(WT) antibody, full-serum) that was preabsorbed either with Arl3-GST (**C1**) or GST only (**D1**) and mouse monoclonal antibodies against RIBEYE(B)/CtBP2 (BD Transduction Labs) (**C2**,**D2**). Signals were overlaid in (**C3**,**D3**). (**E**–**J**) Cryostat sections of wild-type mouse retina double-immunolabeled with rabbit polyclonal antibodies against Arl3(T31N) immune (**F1**) and preimmune (**G1**) serum, affinity-purified rabbit Arl3 antibody (**E1**,**H1**–**J1**) and mouse monoclonal antibodies against SV2 (**E2**) and RIBEYE(B) (clone 2D9) (**F2**–**J2**). Channels are overlaid in (**E3**,**F3**,**G3**,**H3**,**I3**,**J3**). Different secondary antibodies conjugated to different fluorophores were used in (**A**–**D**) vs. (**E**–**H**) to exclude any influence of the secondary antibody and the fluorophore. In (**A**–**D**) Arl3 was visualized with an anti-rabbit secondary antibody conjugated to Alexa568; in (**E**–**F**) Arl3 was visualized with an anti-rabbit secondary antibody conjugated to Alexa488. (**A**–**H**) were obtained by confocal microscopy; (**I**–**J**) by super-resolution structured illumination microscopy (SR-SIM). Abbreviations: ONL, outer nuclear layer; OPL, outer plexiform layer. Scale bars: 3 µm (**A**,**C**,**D**,**E**–**H**); 1 µm (**B**,**I**,**J**).

For the immunolocalization of Arl3 in photoreceptor ribbon synapses in the outer plexiform layer (OPL), we used two independent Arl3 antibodies, i.e., anti-Arl3(WT) and anti-Arl3(T31N) (see Materials and Methods) with similar results. The Arl3(WT) antibody generated strong horseshoe-shaped immunosignals in the OPL of the bovine retina that largely co-localized with synaptic ribbon immunosignals, as judged by double-immunolabeling with anti-RIBEYE (Figure 1A,B). This localization suggested that Arl3 is enriched at the synaptic ribbon in the OPL (Figure 1A,B). Preabsorption experiments were performed to analyze the specificity of the Arl3 immunosignals (Figure 1C,D). Preabsorption of the Arl3(WT) antibody with Arl3-GST (Figure 1C), but not with GST alone (Figure 1D), blocked the Arl3 immunosignal in the OPL, whereas the immunolabeling of RIBEYE was unaffected by the preabsorption (Figure 1C2,D2).

A second rabbit polyclonal Arl3 antibody Arl3(T31N) generated a very similar immunosignal in the OPL of the adult mouse retina (Figure 1E–J). The Arl3(T31N) antibody was raised against a point-mutated Arl3(T31N) protein that mimics the conformation of the GDP-bound form of Arl3 [37,38]. As mentioned in the “Materials and Methods” section, we used a point-mutated version of Arl3 (Arl3(T31N)) for immunization to promote an enhanced antibody response by exposing additional conformational epitopes of Arl3 to the immune system. The Arl3(T31N) antibody detects Arl3 wild-type proteins (with no point mutation) in Western blot analyses and, thus, provided a second, independent antibody capable to detect Arl3 (Supplementary Figure S1C). This second Arl3 antibody Arl3(T31N) also produced a punctate, horseshoe-shaped Arl3 immunosignal in the OPL (Figure 1E). The Arl3 immunosignal was located within the presynaptic terminals that were immunolabeled with anti-SV2 (Figure 1E2). Arl3(T31N) immune serum, but not preimmune serum, produced a strong ribbon-typical immunosignal in the OPL that largely overlapped with the anti-RIBEYE signal. A ribbon-typical Arl3 signal was also observed with the affinity-purified Arl3(T31N) antibody (Figure 1E1,H1) as demonstrated by anti-RIBEYE double-immunolabeling (Figure 1F2,G2,H2). Thus, the enrichment of Arl3 at the synaptic ribbon was consistently observed with both Arl3 antibodies at the light microscopic level. A synaptic ribbon-typical immunostaining pattern of Arl3 was also observed by SR-SIM as judged by double-immunolabeling with Arl3(T31N) and RIBEYE (2D9) antibodies (Figure 1I–J).

We also determined the ultrastructural localization of Arl3 in photoreceptor synapses. The Arl3(WT) antibody was not suitable for ultrastructural analyses. For the ultrastructural localization of Arl3, we used the Arl3(T31N) antibody and pre-embedding immunogold electron microscopy with ultrasmall gold particles conjugated to the secondary antibody, followed by silver enhancement of the ultrasmall immunogold particles.

**Figure 2:**
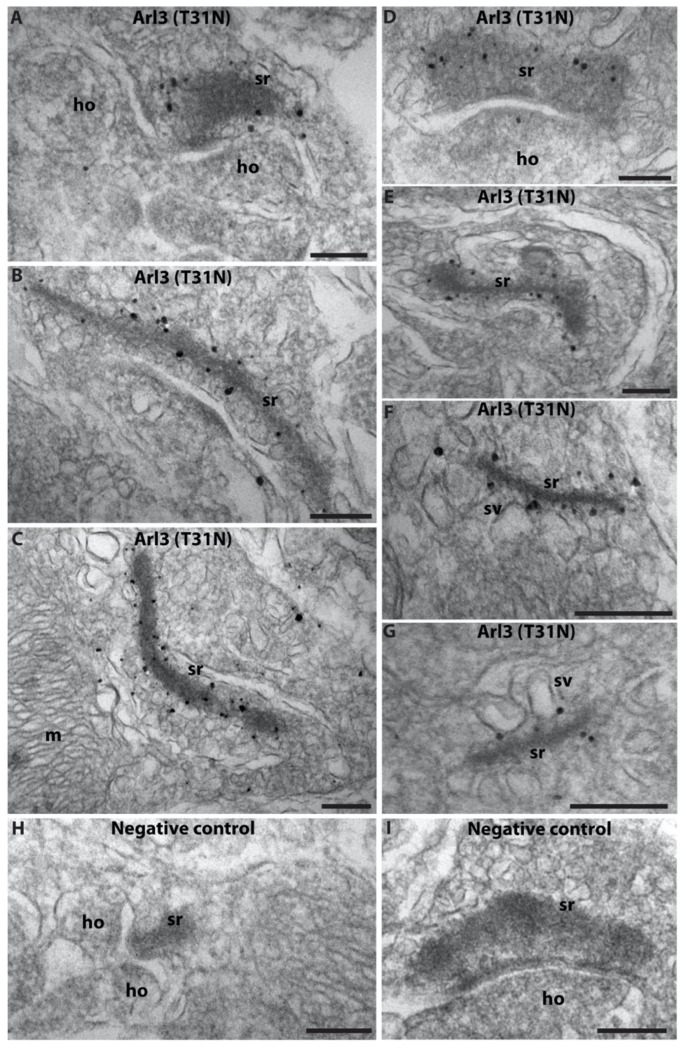
Ultrastructural localization of Arl3 by pre-embedding immunogold electron microscopy. (**A**–**G**) Cryostat sections from wild-type mouse retina were immunolabeled by pre-embedding immunogold microscopy with affinity-purified Arl3(T31N) antibody and silver-enhanced gold nanoparticles conjugated to the secondary antibody. (**H**,**I**) Negative control in which the primary antibody was omitted with all other steps of the procedure being the same. Abbreviations: sr, synaptic ribbon; sv, synaptic vesicles; pre, presynaptic terminal; ho, horizontal postsynaptic dendrites; m, mitochondria. Scale bars: 200 nm (**A**–**I**).

The ultrastructural analyses confirmed the light microscopical immunolabeling data presented above and showed dense Arl3 immunogold signals at the synaptic ribbon of rod photoreceptor synapses (Figure 2).

### 2.2. Rp2 Is Enriched on Synaptic Vesicles in the Presynaptic Photoreceptor Terminal

**Figure 3:**
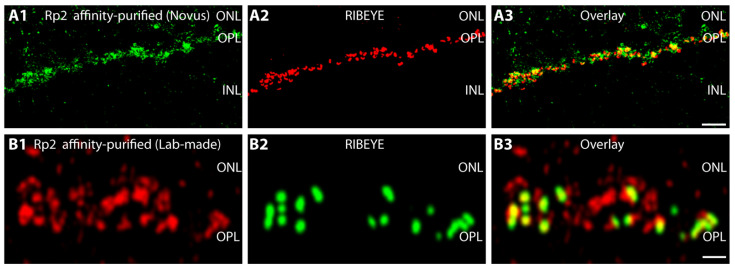

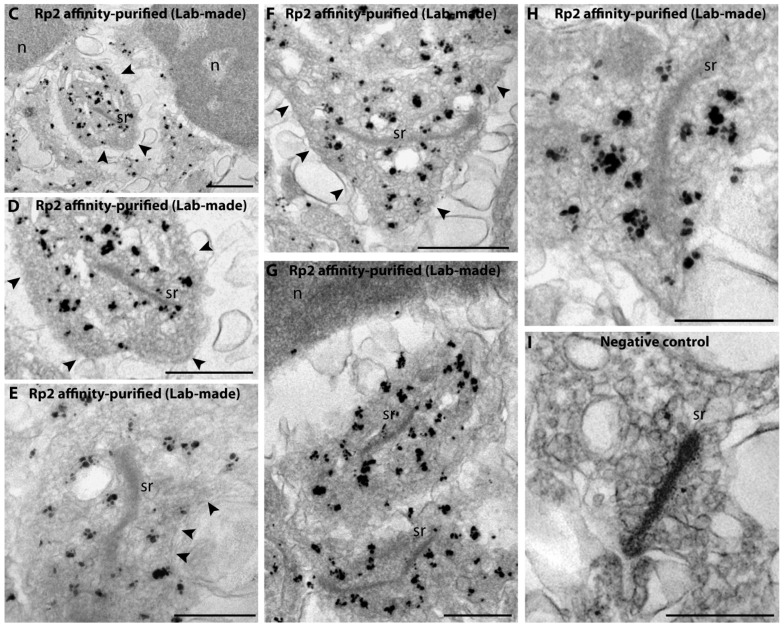
Immunolocalization of RP2 (light and electron microscopy). (**A1**–**A3,B1**–**B3**) Bovine retina sections (**A**: 10 µm-thick cryostat sections; **B**: 0.5 µm-thin resin sections) immunolabeled with the indicated two different anti-Rp2 antibodies (**A1**,**B1**) and anti-RIBEYE(B)/CtBP2 (BD Transduction Labs) (**A2**,**B2**). Signals were overlaid in (**A3**,**B3**). Affinity-purified Rp2 antibody (Novus) was used in (**A**); lab-made, affinity-purified Rp2 antibody in (**B**). RP2 is highly enriched in the OPL that contains the photoreceptor synapses in high density. RP2 immunofluorescence signals surround the immunolabeled synaptic ribbons and are present also at non-ribbon sites. Different secondary antibodies conjugated to different fluorophores were used in (**A**,**B**) to exclude any influence of the secondary antibody and the fluorophore. Micrographs were obtained by confocal microscopy. (**C**–**I**) Pre-embedding immunogold electron microscopy of weakly fixed cryostat sections (from bovine retina) immunolabeled with affinity-purified anti-Rp2. Silver-enhanced RP2 immunogold particles are localized on the bulk of synaptic vesicles in the presynaptic terminal (**C**–**H**). The surrounding plasma membrane, denoted by arrowheads in (**C**–**F**), did not show any RP2 immunolabeling. (**I**) Negative control, in which the primary antibody was omitted with all other parts of the procedure being the same. Abbreviations: ONL, outer nuclear layer; OPL, outer plexiform layer; INL, inner nuclear layer; sr, synaptic ribbon; arrowheads, presynaptic plasma membrane; n, nucleus photoreceptor cell. Scale bars: 5 µm (**A**); 1 µm (**B**); 500 nm (**C**–**I**).

Next, we analyzed the distribution of the Retinitis Pigmentosa 2 (Rp2) protein, the GTPase-activating protein (GAP) of Arl3 [32,33], in the OPL where the photoreceptor synapses are located. With two different, independent antibodies against Rp2, we observed strong Rp2 immunofluorescence signals in the OPL (Figure 3A,B). Both Rp2 antibodies produced very similar immunolabeling patterns. The Rp2 immunosignals were enriched around the synaptic ribbons that were visualized with anti-RIBEYE (Figure 3A2,B2). The Rp2 immunosignals appeared to be less restricted to the synaptic ribbon and showed a broader distribution in the presynaptic terminal than the immunosignals observed for Arl3 (see Figure 1). This observation was supported by pre-embedding immunogold electron microscopy (Figure 3C–H). The silver-enhanced Rp2 immunogold particles were localized on the bulk of synaptic vesicles in the presynaptic terminal (Figure 3C–H). The plasma membrane that surrounds the presynaptic terminal (denoted by arrowheads in Figure 3C–F) did not show any Rp2 immunolabeling. The Rp2 immunolabeling signals were not restricted to synaptic ribbon-associated synaptic vesicles but were also present on synaptic vesicles without association to the synaptic ribbon (Figure 3C–H).

### 2.3. Arl13b Is Enriched on the Synaptic Ribbon in Rod Photoreceptor Terminals

**Figure 4:**
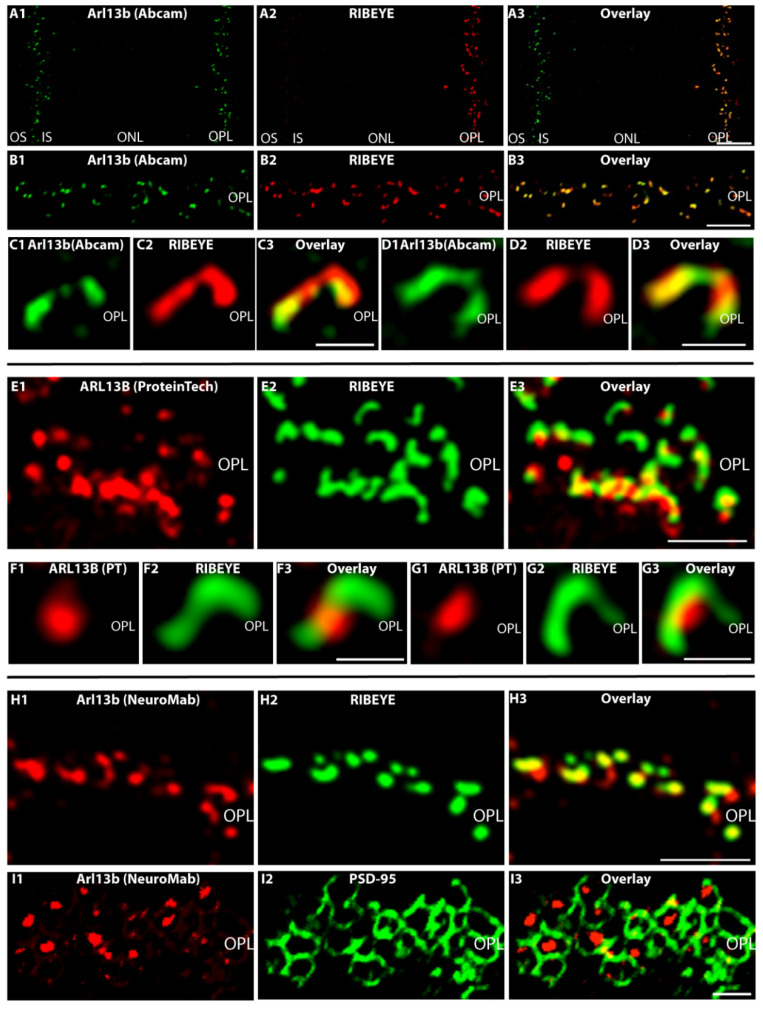
Immunolocalization of Arl13b (light microscopy) (**A**–**I**). (**A1**–**A3**,**B1**–**B3**,**C1**–**C3**,**D1**–**D3**) Cryostat sections of the mouse retina immunolabeled with affinity-purified rabbit polyclonal antibody against Arl13b (Abcam) (**A1**–**D1**) and (**E**–**G**) cryostat section of bovine retina immunolabeled with affinity-purified rabbit antibody against Arl13b (Proteintech, PT) (**E1**–**E3**,**F1**–**F3**,**G1**–**G3**). In addition to immunolabeling of the outer and inner segments, the affinity-purified Arl13b antibodies produced a punctate, horseshoe-shaped immunosignal in the OPL at the ribbons that were labeled with mouse monoclonal antibodies against RIBEYE (2D9) (**A2**–**G2**). (**H**–**I**) Semi-thin resin sections of the mouse retina were immunolabeled with affinity-purified mouse monoclonal antibody against Arl13b (NeuroMab) (**H1**,**I1**), rabbit polyclonal antibody against RIBEYE (U2656) (**H2**) and rabbit polyclonal antibody against PSD-95 (**I2**). Signals are overlaid in (**H3**,**I3**). Please note that different secondary antibodies conjugated to different fluorophores were used in (**A**–**D**) vs. (**E**–**I**) to exclude any influence of the secondary antibody and the fluorophore. Abbreviations: OS, outer segment; IS, inner segment; ONL, outer nuclear layer; OPL, outer plexiform layer. Scale bars: 5 µm (**A**,**B**,**E**,**H**); 1 µm (**C**,**D**,**F**,**G**,**I**).

We used three independent antibodies to determine the localization of Arl13b, the guanine nucleotide exchange factor (GEF) of Arl3, in semi-thin sections and cryosections of the retina by immunofluorescence microscopy (Figure 4). Semi-thin sections were obtained from resin-embedded retinas and sectioned to a thickness of 0.5 µm. With all three antibodies, we consistently observed an enrichment of Arl13b immunosignals at the synaptic ribbon in the photoreceptor synapses of the OPL, as judged by double-immunolabeling with anti-RIBEYE (Figure 4). Arl13b immunosignals were enriched in close vicinity to photoreceptor synaptic ribbons in the OPL (Figure 4A–I and Supplementary Figure S2).

Preabsorption of the Arl13b antibody (Arl13b, Proteintech) performed either with the respective Arl13b fusion protein (Supplementary Figure S2C1) or with the control protein (Supplementary Figure S2A1,B1) documented the specificity of the immunolabeling data (Supplementary Figure S2B1,B3,C1–C3). Preabsorption with Arl13b-GST abolished the Arl13b immunosignal in the OPL, whereas preabsorption of the Arl13b antibody with the control protein had no impact on the Arl13b signal in the OPL. Co-staining with RIBEYE antibodies was unaffected in both preabsorption experiments (Supplementary Figure S2A2,B2,C2). Similarly, the Arl13b immunosignal in the OPL generated by the Arl13b Abcam antibody was efficiently blocked by the respective peptide but not by a control peptide (Supplementary Figure S2D–E). Arl13b appeared to be also present in the less abundant cone synapses as judged by double-labeling with anti-Arl13b and fluorescent PNA lectin (Supplementary Figure S2F,G).

**Figure 5:**
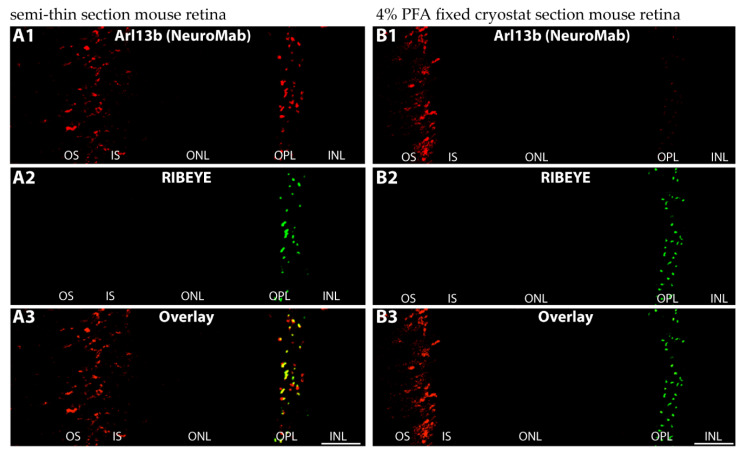
Synaptic Arl13b immunosignals are dependent upon fixation. Semi-thin sections (**A1**–**A3**) (not fixed with PFA) and cryostat section (**B1**–**B3**) obtained from mouse retina that has been fixed with 4% PFA were immunolabeled with affinity-purified mouse monoclonal antibody against Arl13b (NeuroMab) (**A1**,**B1**) and rabbit polyclonal antibody against RIBEYE (U2656) (**A2**,**B2**). Abbreviations: OS, outer segment; IS, inner segment; ONL, outer nuclear layer; OPL, outer plexiform layer; INL, inner nuclear layer. Scale bars: 5 µm (**A**,**B**).

**Figure 6:**
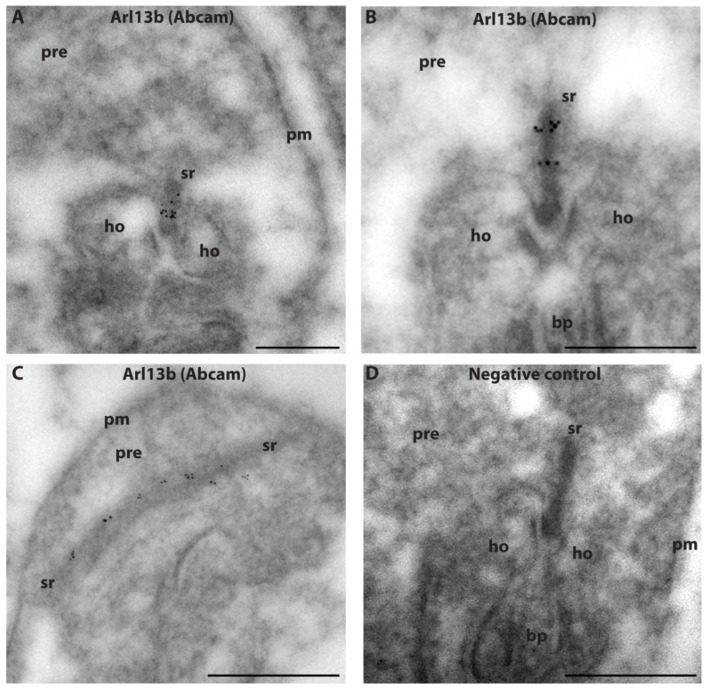
Ultrastructural localization of Arl13b (**A**–**C**). Post-embedding immunogold labeling of rod photoreceptor synapses (mouse retina) with affinity-purified Arl13b (Abcam) antibody. (**D**) shows a negative control. Abbreviations: sr, synaptic ribbon; pre, presynaptic terminal; ho, horizontal cell postsynaptic dendrites; bp, bipolar cell postsynaptic dendrites; pm, plasma membrane. Scale bars: 500 nm (**A**–**D**).

The monoclonal Arl13b antibody (NeuroMab) was previously used for immunolocalization of Arl13b in the retina by using cryostat sections of a paraformaldehyde (PFA)-fixed mouse retina [39]. No Arl13b synaptic signal was observed in the OPL with this method [39]. We confirmed the absence of Arl13b immunosignals in PFA-fixed sections of the retina, whereas the same antibody generated a strong synaptic signal in the OPL of semi-thin retina sections of the mouse retina (Figure 4H–I and Figure 5A–B). From this finding we conclude that the synaptic Arl13b is sensitive to PFA fixation and not accessible in PFA-fixed samples. The semi-thin resin method does not employ PFA fixation, but cryofixation, and can provide the antibody easier access to the synaptic antigen. This proposal is in agreement with previous observations that demonstrated that ciliary proteins such as Arl13b are highly sensitive to fixation [40,41]. In conclusion, the light microscopical immunostaining data that were consistently obtained with three independent Arl13b antibodies on semi-thin resin sections and chemically non-fixed cryostat sections provided evidence for localization of Arl13b at the synaptic ribbon complex, similar to what was observed for Arl3. This suggestion was further supported by immunogold electron microscopical analyses. Post-embedding immunogold electron microscopy with the Arl13b antibody (Abcam) revealed a clear Arl13b immunogold signal at photoreceptor synaptic ribbons (Figure 6A–C) that was absent in the control incubations (Figure 6D). [41] also found an enrichment of Arl13b in the OPL, but could not resolve the ultrastructural distribution.

### 2.4. Nphp3 (Nephrocystin-3), a Disease-Relevant Client Protein of Unc119, Is Highly Enriched at the Synaptic Ribbon, Particularly at the Active Zone

**Figure 7:**
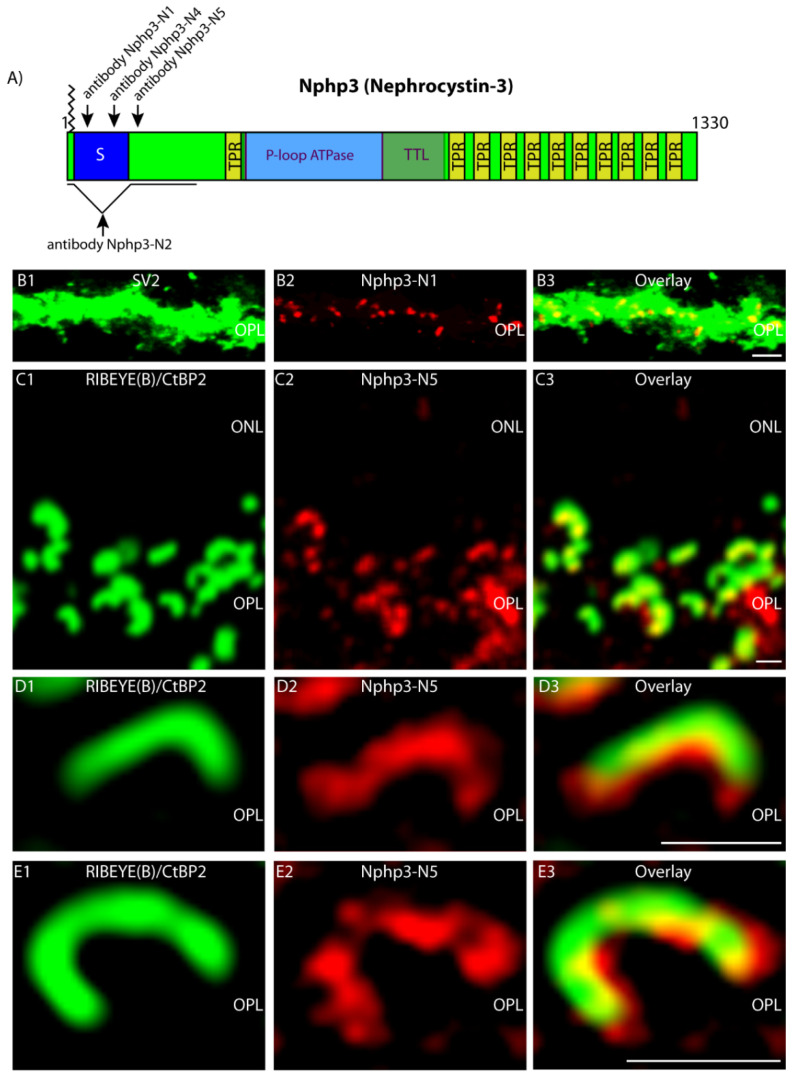
Immunolocalization of Nphp3 (light microscopy). (**A**) Schematic depiction of Nphp3 domain structure. Sequence stretches representing the antigens to which antibodies Nphp3-N1, Nphp3-N2, Nphp3-N4 and Nphp3-N5 have been raised are indicated (arrows). Abbreviations: S, N-terminal alternatively spliced exon; TPR, tetratricopeptide repeat; TTL, tubulin-tyrosine ligase domain. Nphp3 is myristoylated at the N-terminus (aa 2). (**B1**–**B3**) Cryostat section of the mouse retina immunolabeled with mouse monoclonal SV2 antibody (**B1**) and rabbit polyclonal Nphp3-N1 antibody (**B2**). (**C1**–**C3,D1**–**D3,E1**–**E3**) Cryostat section of bovine retina immunolabeled with anti-Nphp3-N5 (clone 5A3) (**C2**,**D2**,**E2**) by indirect immunofluorescence microscopy. Synaptic ribbons were visualized by fluorescently labeled primary mouse monoclonal antibody against RIBEYE(B)/CtBP2 (**C1**,**D1**,**E1**). The Nphp3-N5 antibody produced punctate, horseshoe-shaped immunosignals in the OPL where the photoreceptor synapses are located, overlapping with the ribbons. Nphp3-N1, Nphp3-N2 and Nphp3-N4 antibodies also generated very similar immunosignals in the OPL (Supplementary Figures S3, S5 and S6). (**B**,**C**) were obtained by confocal microscopy; (**D**,**E**) by SR-SIM. SR-SIM data (**D**,**E**) show an enrichment of Nphp3 at the base (concavity) of the horseshoe-shaped synaptic ribbon. Abbreviations: ONL, outer nuclear layer; OPL, outer plexiform layer. Scale bars: 5 µm (**B**); 1 µm (**C**–**E**).

Nphp3 (Nephrocystin-3), a protein associated with Senior–Løken Syndrome 3 (SLS3) [42,43], is a well-known myristoylated multidomain client protein (Figure 7A) of Unc119 at the photoreceptor primary cilium [9]. Therefore, we tested whether Nphp3 is also present in the photoreceptor synapse at the synaptic ribbon. We addressed this question using four independent antibodies against Nphp3 (denoted as antibody Nphp3-N1, Nphp3-N2, Nphp3-N4, Nphp3-N5) raised against different peptide regions of the protein (Figure 7A). All antibodies were characterized by Western blot on retinal lysates, transfected HEK293T cells or overlapping peptide spot arrays to verify the specificity of the antibodies (Supplementary Figures S3A–F and S4). The antibodies showed a single immunoreactive band at the expected running position (Supplementary Figure S3B–F). The pepspot arrays revealed the epitopes in the alternatively spliced exon of Nphp3, detected by the Nphp3-N1 and Nphp3-N4 antibodies (Supplementary Figure S4).

Next, we applied these antibodies in light microscopical immunolabeling analyses. All four different Nphp3 antibodies consistently demonstrated a strong enrichment of Nphp3 in photoreceptor presynaptic terminals (Figure 7B–E, Figures S3G, S5 and S6). This was shown for all Nphp3 antibodies, both by high-resolution confocal microscopy (Figure 7B,C, Figure S3G and S5) as well as by super-resolution structured illumination microscopy (SR-SIM) (Figure 7D,E and Figure S6). All antibodies demonstrated an enrichment of Nphp3 at the synaptic ribbon that was immunolabeled with RIBEYE antibodies (Figure 7B–E, Figure S3G, S5 and S6A). Further double-immunolabeling analyses (Supplementary Figure S6B–D) revealed that Nphp3 immunosignals largely overlapped with the immunosignals for RIM1/2, a key component of the photoreceptor active zone complex [28].

**Figure 8:**
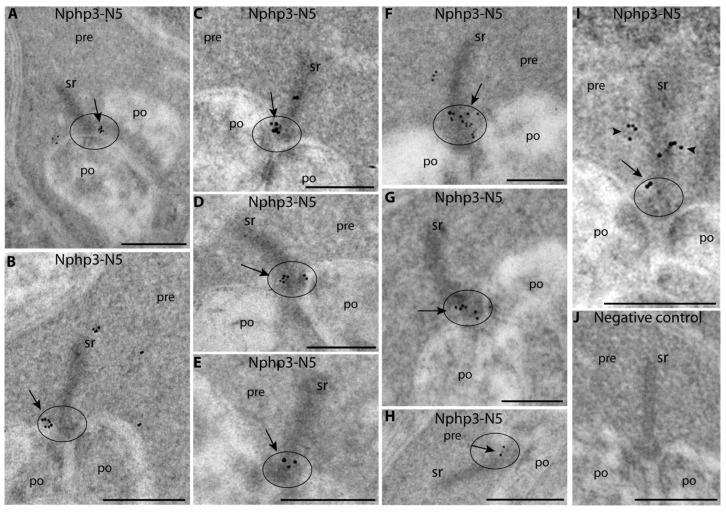

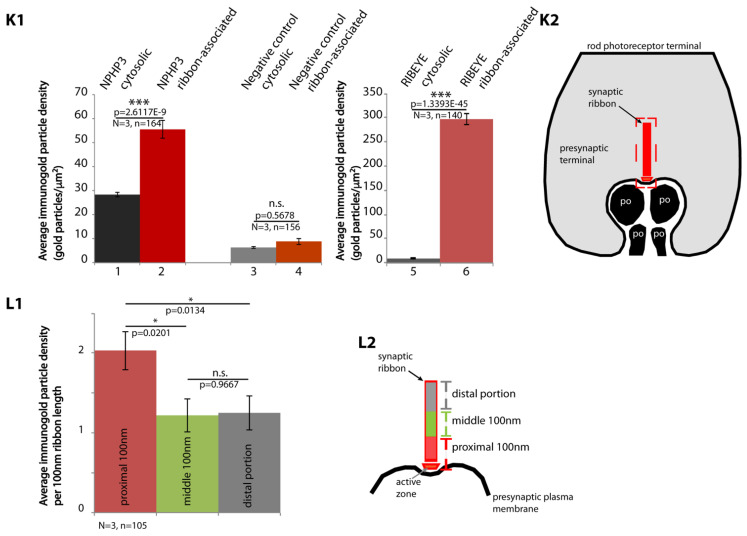
Ultrastructural localization of NPHP3 in rod photoreceptor synapses. (**A**–**I**) Post-embedding immunogold labeling of rod photoreceptor synapses (bovine retina) with mouse monoclonal anti-NPHP3 (Nphp3-N5). NPHP3 immunogold particles are enriched at the synaptic ribbon, particularly at the base of the ribbon, at the active-zone-anchored proximal end of the ribbon (arrows in **A**–**I**). Abbreviations: sr, synaptic ribbon; pre, presynaptic terminal; po, postsynaptic dendrites. (**J**) is a negative control for which no primary antibody, but only secondary antibody, was used. (**K1**) Quantification of the post-embedding immunogold labeling data. Average immunogold puncta density at the synaptic ribbon (bar 2) was compared to average immunogold puncta density in the presynaptic terminal at non-ribbon-containing cytosolic sites (bar 1). Bars 3 and 4 summarize quantification of the background immunogold particle densities at the synaptic ribbon and non-ribbon cytosolic sites in the rod photoreceptor terminal. Bars 5 and 6: distribution of RIBEYE immunogold puncta (positive control) at the synaptic ribbon and at non-ribbon sites. In (**K2**), the respective areas analyzed for the determination of immunogold particle density are schematically depicted; red, the synaptic ribbon; grey, presynaptic terminal. Scale bars: 500 nm (**A**–**J**). In (**L1**), the distribution of NPHP3 immunogold particles were quantified at the indicated regions of the synaptic ribbon. The analyzed portions of the synaptic ribbon are summarized in (**L2**). Error bars are s.e.m. Number of experiments and actual *p*-values are plotted in the figure.

The monoclonal Nphp3-N5 antibody against Nphp3 was suitable for post-embedding immunogold electron microscopy. Post-embedding immunogold labeling confirmed the light microscopical results and demonstrated the precise, ultrastructural localization of Nphp3 at photoreceptor ribbon synapses. The ultrastructural analyses demonstrated that Nphp3 is enriched at the synaptic ribbon of photoreceptor synapses (Figure 8A–I; Figure 8J is a negative control). A minor portion of Nphp3 immunosignals was also found in some distance to the synaptic ribbon (Figure 8A–I, quantitative assessment in Figure 8K). At the synaptic ribbon, the majority of the Nphp3 immunogold signal was present at the base of the synaptic ribbon, in close vicinity (within a 100 nm distance) to the presynaptic plasma membrane of the active zone that also includes the proper active zone (arrows in Figure 8A–I; for quantification, see Figure 8L).

### 2.5. The Synaptic Ribbon Enhances Synaptic Enrichment of Nphp3

**Figure 9:**
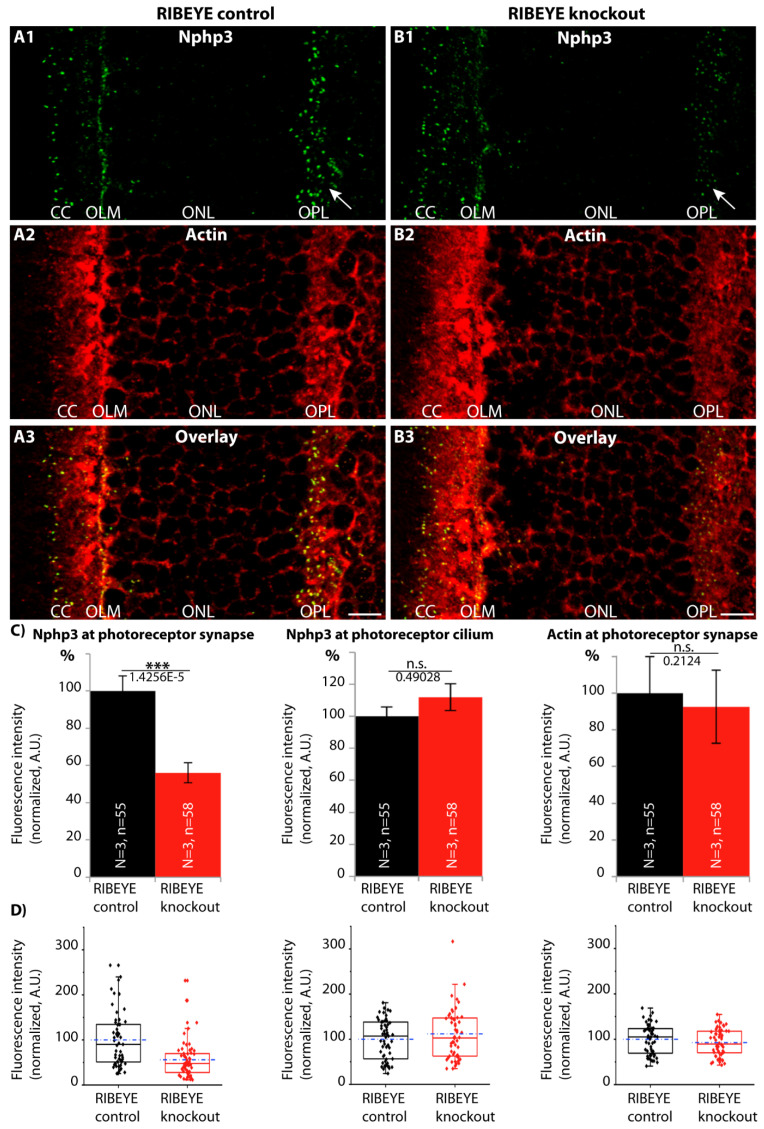
Synaptic enrichment of Nphp3 to active zones is facilitated by the synaptic ribbon. (**A1**–**A3,B1**–**B3**) Cryostat sections of RIBEYE knockout mice (**B1**–**B3**) and control mice (**A1**–**A3**) immunolabeled with the indicated antibodies. Arrows in (**A1**,**B1**) point to synaptic Nphp3 immunosignals in the OPL of the indicated genotypes. In (**C**,**D**), the Nphp3-N1 immunosignals at the synapse and the connecting cilium and the actin immunosignal at the synapse were quantified. In (**C**), the quantifications are displayed as bar diagrams. Error bars are s.e.m. In (**D**), the individual values are displayed as box-and-whiskers diagrams. Dashed blue lines in (**D**) are mean values, solid black lines in the boxes show the respective median values; boxes and whiskers illustrate 25–75 and 5–95 percentiles of values, respectively. Number of experiments and analyzed sections, together with the actual *p*-values, are directly plotted in the graphs in (**C**). Statistical significance was determined with a Mann–Whitney U test (Origin Pro). Abbreviations: CC, connecting cilium; OLM, outer limiting membrane; ONL, outer nuclear layer; OPL, outer plexiform layer. Scale bars: 5 µm (**A**,**B**).

Since we found Nphp3 being associated with the synaptic ribbon, particularly with the basal end of the ribbon that is anchored to the active zone (Figure 8), we asked whether the synaptic localization of Nphp3 is dependent upon the presence of the synaptic ribbon. For this purpose, we analyzed the distribution of Nphp3 in the recently generated RIBEYE knockout mouse in which synaptic ribbons are selectively absent [30]. We found that the degree of synaptic localization of Nphp3 is strongly reduced in the RIBEYE knockout mouse (Figure 9), suggesting that the synaptic ribbon is needed for the efficient transport of Nphp3 to its synaptic localization at the photoreceptor active zone.

## 3. Discussion

In the present study, we demonstrated that a set of “ciliary” proteins involved in the transport of lipid-modified proteins is present also at the synaptic ribbon complex of rod photoreceptor synapses. These proteins include Arl3, Rp2 and Arl13b that control the binding and discharge activity of Unc119 for lipid-modified protein cargo. Unc119 was previously shown to be enriched in photoreceptor ribbon synapses [19,20,21] and functions as a GDI-like carrier protein for myristoylated lipid-modified proteins [11,12,14,15].

The “discharge” activity of Unc119 for myristoylated proteins is regulated by Arl3 and the nature of the bound guanine nucleotide. Arl3-GTP promotes the discharge of lipidated protein cargo into target membranes. Conversely, Rp2 enhances the intrinsically low GTPase activity of Arl3 and the resulting Arl3-GDP recycles the Unc119 complex for a new round of binding [6]. Arl3-GDP is reactivated by Arl13b, the guanine nucleotide exchange factor (GEF) for Arl3, to restore Arl3-GTP. This Unc119-dependent binding/release cycle is accompanied also by trafficking of Unc119 from one membrane compartment to another [6,15].

In the present study, we demonstrated that the synaptic ribbon complex of rod photoreceptor synapses is enriched in these regulatory proteins that control Unc119 function. We showed that Arl3 and Arl13b are directly localized at the synaptic ribbon by high resolution immunofluorescence microscopy using multiple independent antibodies. These independent antibodies consistently confirmed localization of Arl3 and Arl13b at the synaptic ribbon. The localization of Arl3 and Arl13b to the synaptic ribbon was also confirmed by immunogold electron microscopy at the ultrastructural level. Since these GTP-binding proteins Arl3/Arl13b are largely “discharge factors” for lipid-modified cargo, we speculate that Arl3/Arl13b promote the discharge of lipid-modified protein cargo at the synaptic ribbon.

High-resolution immunofluorescence microscopy also demonstrated the presence of Rp2 at photoreceptor ribbon synapses, but not as close to the synaptic ribbon as Arl3 and Arl13b. Indeed, our ultrastructural analyses revealed that Rp2 is not located at the ribbon itself but on synaptic vesicles that surround the synaptic ribbon (Figure 3C–H). At that site, Rp2 could prevent a premature release of lipidated protein cargo from Unc119 by keeping Arl3 in its inactive, GDP-bound form when it is not at the ribbon. Considering that fact, Rp2 would prevent a premature discharge of lipid cargo at the wrong subcellular site. A synaptic localization was also previously observed for Rp2 at the light microscopic level [44,45,46]. However, the ultrastructural distribution could not be resolved in these earlier studies.

Arl3/Arl13b and Rp2 are responsible for the cycling of Unc119 between a cargo-loaded and cargo-discharged state to promote trafficking of the lipid-modified protein complex between membrane compartments (for review, [6,47]). Since this set of Unc119-associated proteins present at the ribbon complex is well-known to be responsible for the shuttling of lipid-modified proteins at the cilium [6,14,15,31,48]), our data propose that these proteins perform a similar function in mediating translocation of lipid-modified proteins also at the synaptic ribbon.

Surprisingly, at least one cargo protein of Unc119 appears to be conserved between the photoreceptor primary cilium and the photoreceptor synapse, i.e., Nphp3. We demonstrated that Nphp3, a myristoylated, Unc119-dependent cargo protein at the connecting cilium, is also present at the synaptic ribbon. The localization of Nphp3 at the synaptic ribbon is new and surprising. Several independent antibodies directed against regions of Nphp3 confirmed the localization of Nphp3 at the synaptic ribbon as judged by high-resolution, light-microscopical immunolabeling techniques. Furthermore, post-embedding immunogold electron microscopy also demonstrated the enrichment of Nphp3 at the synaptic ribbon. Interestingly, Nphp3 was particularly enriched at the basal membrane-proximal end of the synaptic ribbon that also includes the proper active zone. This was shown by post-embedding immunogold electron microscopy and quantitative analyses of the Nphp3 immunogold particles. Thus, Nphp3 can be considered as a novel component of the cytomatrix of the active zone of photoreceptor ribbon synapses.

The transport of Nphp3 to the active zone is dependent upon the presence of the synaptic ribbon because its synaptic localization is strongly reduced in RIBEYE knockout mice. In RIBEYE knockout mice, we observed a significantly decreased enrichment of Nphp3 in the synapse but not at the connecting cilium, demonstrating the specificity of the synaptic phenotype. The function of Nphp3 at the photoreceptor active zone remains to be elucidated. Nphp3 is a multidomain protein including a coiled-coil domain, tubulin-tyrosine-ligase domain and several TPR repeats [42]. Recently, it was observed that pcy mice, that possess a spontaneous mutation in the *Nphp3* gene, show aberrant expression of the Rap1-/Rab27-binding, C2 domain-containing synaptotagmin-like protein 2 (Slp2-a) in renal cells [49]. These proteins are involved in targeted membrane transport and in the generation of specialized docking sites [50,51]. Similar mechanisms might be installed at the photoreceptor ribbon synapse. Clearly, future investigations are needed to address the function of Nphp3 at the synapse. Of note, mutations in the Nphp3 gene are associated with Senior–Løken Syndrome 3 (SLS3) characterized by retinal degeneration and vision loss [42,43]. Thus, vision loss in SLS3 in humans might not only be based on ciliary dysfunctions, but also on malfunctions of the photoreceptor synapse.

Interestingly, several other proteins also share a dual localization at the photoreceptor cilium and the photoreceptor synaptic ribbon. These include the PIP_2_-binding tubby-like protein 1 (Tulp1) that is present both at the photoreceptor synaptic ribbon complex [52,53,54] and the photoreceptor cilium [52,54]. The same dual localization, i.e., at the cilium and the ribbon, has been also described for the kinesin-2 motor protein Kif3a [55,56,57,58,59]. Similarly, the ciliary protein Nphp4 is important for normal ribbon synapse maintenance, as shown by knockout analyses [60]. Thus, the photoreceptor synaptic ribbon appears to have several components in common with the primary cilium, raising the possibility that common functional mechanisms could also prevail at these two compartments. In agreement with this proposal, the t-SNARE protein Syntaxin-3 is essential for vesicle fusion both at the photoreceptor cilium as well as at the synaptic ribbon [61,62,63,64]. Future analyses might reveal further molecular and functional similarities between the synaptic ribbon and primary cilia.

## 4. Materials and Methods

### 4.1. Animals

Experiments were performed on tissues obtained from C57BL/6J mice of both sexes and bovine retinas as indicated in the respective experiments. Retinas from two species were used to exclude the possibility that the observed findings might be species-specific. Animal care and all experimental procedures that involved mice were performed according to the guidelines of the German Animal Protection Law (Tierschutzgesetz) and were reviewed and approved by the animal welfare and ethics committee of Saarland University and the local authorities (Landesamt für Verbraucherschutz; Geschäftsbereich 3; 66115 Saarbrücken, Germany; GB 3-2.4.1.1-K110/180-07). Mice were kept under standard light/dark cycle and supported with standard food and water ad libitum. Mouse retinas were obtained from the indicated mice (3–6 months of age) within 5 min post mortem, as previously described [29,53,65,66,67]. RIBEYE knockout mice (Ctbp2^tm1.2Sud^) were previously generated and characterized [30]. Bovine retinas were obtained from a local slaughterhouse.

### 4.2. Primary Antibodies

#### 4.2.1. Arl3

Arl3 is a small (182 aa in *Rattus norvegicus*; NP_073191.1), GTP-binding ARF-like protein [13,14]. The GTP-bound form of Arl3 is a release factor for Unc119-dependent lipidated protein cargo [9,11]. The GTPase activity of Arl3 is enhanced by the Retinitis Pigmentosa 2 (Rp2) protein that is a GTPase-activating protein (GAP) for Arl3 [14,36]. Arl13b is a GTP exchange factor (GEF) protein that stimulates exchange of GDP from inactive Arl3 by GTP to activate Arl3. At the light microscopical level, Arl3 was already shown to localize to the photoreceptor connecting cilium, the inner-segment cell body and synaptic terminal in photoreceptors [44,68]. Its ultrastructural localization in the synaptic terminal has not been determined.

We used two different, self-made rabbit polyclonal antibodies against Arl3.

- Anti-Arl3(WT): Lab-made rabbit polyclonal antiserum against full-length rat Arl3. The antiserum was raised against a pGEX fusion protein construct encoding aa1-182 of rat Arl3. The full-length insert was cloned via *NcoI*/*SalI* in frame into the respective sites of pGEX-KG. The antiserum was used in a 1:100 dilution for IF and in a 1:500 dilution for WB. The affinity-purified antibody was used in a 1:50 dilution for IF and in a 1:100 dilution for WB.
- Anti-Arl3(T31N): Lab-made rabbit polyclonal antiserum against a point-mutated Arl3(T31N)-MBP fusion protein. The Arl3(T31N) antibody also detects wild-type Arl3 protein (see Supplementary Figure S1). The T31N point mutant of Arl3 mimics the conformation of GDP-bound Arl3 [37,38]. We used the Arl3(T31N) point mutant for immunization because we wanted to elicit an enhanced antibody response against Arl3 by exposing additional conformational epitopes of Arl3 to the immune system. It is known that Arl3 is a difficult protein for the generation of antibodies [68]. With the described strategy of using a point-mutated protein variant, we wanted to increase the likelihood of generating a good polyclonal antibody response against Arl3. Indeed, with this strategy we obtained an antibody that readily detected the Arl3 wild-type protein in Western blot analyses (Supplementary Figure S1). Therefore, we used this antibody as a second independent antibody for the localization of Arl3. For our present study, it is not relevant whether the antibody would also detect the T31N point mutation because we only analyzed wild-type retinas.

**Generation of the Arl3(T31N) point mutation.** The point-mutated rat Arl3(T31N) bacterial expression plasmid was generated by site-directed mutagenesis. The T31N point mutation was first introduced into a wild-type Arl3-mCherry construct encoding aa 1–182 of rat Arl3 (glycine at position 2) via PCR with forward primer GACAACGCTGGCAAGAACACGTTGCTGAAGCAGCTTG and reverse primer CAAGCTGCTTCAGCAACGTGTTCTTGCCAGCGTTGTC (underlined is the point-mutated codon) using PrimeStar HS DNA Polymerase (Takara, Saint-Germain-en-Lave, France, #R010a). The PCR product was purified with the QIAquick PCR Purification Kit (Qiagen 28104). The *DpnI* restriction endonuclease (NEB, Frankfurt, Germany, R0176S) was used to remove original template plasmid from PCR product. DNA was ethanol precipitated and transformed in chemically competent DH10B bacteria. Candidate clones for the amino acid substitution were checked by restriction analysis of plasmid DNA for the silent restriction site *AflIII* (NEB, Frankfurt, Germany, R0541S) present in the point-mutated sequences. Positive clones were further verified by Sanger sequencing.

**Cloning of Arl3(T31N) in pMalC2.** The point-mutated Arl3(T31N) construct was PCR amplified with forward primer AAAAGAATTCATGGGCTTGCTCTCTATTTTG; the reverse primer AAAAGTCGACTTACTTCTTCTTTGCGTTGAC using the Arl3(T31N)-mCherry plasmid as template. The PCR product was cloned into the *EcoRI*/*SalI* sites of pMalC2 and verified by sequencing [66]. A fusion protein was expressed and purified using standard methods [66] and the eluted, purified fusion protein was used to immunize a rabbit (five immunizations, 100 µg each). The antiserum was used in a 1:500 dilution for IF and in a 1:2000 dilution for WB. The affinity-purified antibody was used in a 1:100 dilution for IF and 1:500 for WB, and 1:100 dilution for pre-embedding immunogold electron microscopy from a 1 mg/mL concentrated stock solution of the affinity-purified antibody (affinity-purified on antigen).

#### 4.2.2. Anti-Rp2 Antibodies

The Retinitis Pigmentosa 2 (Rp2) protein is the GTPase-activating protein (GAP) for Arl3 (350 aa, dually acylated with sites for N-terminal palmitoylation and myristoylation [36,69]). At the light microscopical level, Rp2 was localized close to the basal body of the connecting cilium in photoreceptors and also to the synaptic regions [46].

We used two independent rabbit polyclonal antibodies against RP2.

- Anti-Rp2 (lab-made rabbit polyclonal antiserum against full-length bovine RP2): The corresponding cDNA insert was amplified using forward primer AAAAGAATTCATGGGCTGCTTCTTCTCC, reverse primer AAAAGGATCCT CATATTCCCATCTGTATATC and Image clone 8433421 (BC153222; bovine RP2) as the template. The cDNA construct was cloned into the *EcoRI*/*BamHI* sites of pMalC2New [66]. The antibody was used for IF in a 1:100 dilution, for WB in a 1:500 dilution and for pre-embedding immunogold EM in a 1:100 dilution.
- Anti-Rp2 (Novus, Wiesbaden, Germany, NBP1-56852, affinity-purified rabbit polyclonal antibody): Raised against the amino acid sequence LEFNGDGAVEVCQLIVNEIFNGTKMFVSESK ETASGDVDSFYNFADIQMG, this is an internal peptide region in the middle of human RP2. This antibody was used for IF in a 1:50 dilution and for WB in a 1:1000 dilution.

#### 4.2.3. Anti-Arl13b Antibodies

Arl13b is the GTP/GDP exchange factor (GEF) for Arl3 leading to the formation of the activated Arl3-GTP from Arl3-GDP. For immunolocalization of Arl13b, we used three different commercially available Arl13b antibodies:

- An affinity-purified rabbit anti-Arl13b antibody (Proteintech; Planegg-Martinsried, Germany, 17711-1-AP) raised against the Arl13b-GST fusion protein (Ag12015, Proteintech), encoding full-length human ARL13B (encoding aa 1–321 of BC094725/NP659433 (full-length human ARL13B protein); amino acid sequence 86% identical to bovine ARL13B sequence XP_003585676.1; 75% identical amino acids to mouse Arl13b XP_006522568.1). The antibody was previously characterized (e.g., [39,70,71]) and verified for specificity on the mouse Arl13b knockout tissue by IF and WB [39]. This antibody was used for IF in a 1:50 dilution and for WB in a 1:1000 dilution. For preabsorption control experiments, the antibody was preabsorbed with the respective Arl13b-GST fusion protein that was used for immunization (Arl13b-GST fusion protein obtained from Proteintech (#Ag12015)). Further preabsorption control experiments were conducted with GST alone to check for possible non-specific preabsorption (see below).
- An immunogen affinity-purified rabbit polyclonal anti-Arl13b from Abcam (Cambridge, UK, ab83879) raised against a synthetic peptide aa 251–300 of human ARL13B (NP_659433; VEPLNIDDCAPESPTPPPPPPPVGWGTPKVTRLPKLEPLGETHHN DK). This antibody was used for IF in a 1:500 dilution, for post-embedding immunogold in a 1:100 dilution and for WB in a 1:500 dilution.
- A monoclonal Arl13b antibody (NIH NeuroMab/UC Davis, anti-Arl13b, Clone N295B/66) distributed by Antibodies-online, Aachen, Germany. This monoclonal antibody was raised against a fusion protein encoding amino acids 208–427 of the carboxyterminal half of mouse ARL13B (Q640N2, sequence identity in this stretch in comparison to bovine sequence 72%: XP_003585676). The specificity of this antibody was verified on knockout tissue by NeuroMab (documented on NeuroMab Clone N295B/66 datasheet). In WB analyses, this antibody selectively detects an ≈60 kDa band that is absent in the Arl13b knockout (datasheet NeuroMab).

#### 4.2.4. Nphp3 (Nephrocystein-3)

Nphp3 is a large (1330 amino acids in human NPHP3; AAP83423.1) multidomain-containing protein with several splice variants being deposited in the databases. Nphp3 interacts with Unc119 in a myristoylation-dependent manner [9].

Nphp3 antibodies: we used four independent antibodies raised against different epitopes of Nphp3, which are explained in detail together with their abbreviations.

- Anti-Nphp3-N1: Rabbit polyclonal antibody raised against a peptide region covering aa 1–131 of human NPHP3 (AAP83423.1; Proteintech, Planegg-Martinsried, Germany; #22026-1-AP). This epitope corresponds to the first 7 aminoterminal amino acids (aa), followed by the peptide region encoded by the aminoterminal alternatively spliced exon (aa 8-131) of Nphp3 highlighted in Figure 7A (S). This antibody was previously characterized in WB and IF in human and mouse tissues [72,73]. This antibody was used for IF in a 1:200 dilution, for WB in a 1:500 dilution and for “pepspots“ analyses in a 1:1000 dilution.
- Anti-Nphp3-N2 (lab-made rabbit polyclonal antibody): The antibody was raised against a peptide region comprising aa 1–180 of mouse Nphp3 (AAI15725.1, BC115724). From this cDNA clone, the N-terminal exon indicated in Figure 7A is spliced out. The corresponding cDNA was amplified by PCR using forward primer (AAAATCTAGAATGGGCACAGCCTCGTCG, *XbaI*), reverse primer (AAAAGTCGACAAGGTAGCACCTG ACAGG, *SalI*) and the mouse cDNA Image clone 40086844 (AAI15725.1, BC115724) as the template. The PCR product was cloned into pMalC2New [66], and the fusion protein was purified using standard techniques. This aminoterminal stretch is highly conserved (91% identical aa in mouse Nphp3 (AAI15725.1, BC115724) and bovine NPHP3 (XP_019817929.1)). This antibody was used for IF in a 1:100 dilution, for WB in a 1:500 dilution and for HEK293T cell WB transfection analyses in a 1:1000 dilution.
- Anti-Nphp3-N4 (commercial Biorbyt [#orb221817] via Biozol; affinity-purified rabbit polyclonal antibody): The sequence against which the antibody was raised was not published. This antibody did not detect heterologously expressed Nphp3 (AAI15725.1/BC115724) with a spliced-out N-terminal exon (1204 aa in length) in WB (data not shown). Therefore, we assumed that this antibody is directed against a peptide sequence within the alternatively spliced exon and confirmed this assumption by overlapping peptide arrays (Supplementary Figure S4). The antibody was used for IF in a 1:500 dilution and for pepspot immunoblotting in a 1:2000 dilution.
- Anti-Nphp3-N5: Mouse monoclonal Nphp3-N5 antibody (clone 5A3; IgA immunoglobulin) raised against the peptide sequence ENEIQDLLRAKRELESKLQRLQAQG and corresponding to aa 181–205 of bovine NPHP3 (XP_019817929.1) that is identical in mouse, rat, pig and human NPHP3. This amino acid sequence is located downstream of the alternatively spliced N-terminal exon (see Figure 7A) and is present both in the long and the short Nphp3 isoform. Mouse immunization, generation of hybridoma cells and ELISA selection of hybridoma cells, and antibody subtyping was performed by Absea, Beijing, China. This antibody was used for IF in a 1:200 dilution (corresponding to an immunoglobulin concentration of ≈0.5 µg/mL) and for WB in a 1:500 dilution (corresponding to an immunoglobulin concentration of ≈0.2 µg/mL) (Table 1 and Table 2).

### 4.3. Nphp3 Eukarzyotic Expression Plasmid

For heterologous expression in HEK293T cells, full-length mouse Nphp3 cDNA was amplified from EST clone BC115724 (AAI15725.1) and cloned into the *EcoRI*/*SalI* sites of pCMV-Tag2b. This, and all other plasmid constructs used in the study, were verified by sequencing. HEK293T cells were cultured in DMEM supplemented with 10% fetal calf serum (FCS) and transfected with standard methods.

### 4.4. Various Reagents

Lectin PNA conjugated to Alexa568 (Invitrogen; Karlsruhe, Germany; L32458).

## 5. Methods

### 5.1. Affinity Purification of Antibodies

Affinity purification of most antibodies was performed according to [79]. For the affinity purification of Arl3 antibody, we used glutathione beads to which the GST-tagged Arl3 fusion protein was still bound and cross-linked to glutathione with glutaraldehyde. For this purpose, 50 µg of Arl3-GST fusion proteins were fixed with 1% glutaraldehyde in PBS for 10 min (on ice). Next, fusion protein beads were washed three times with PBS and several rounds of resuspension in PBS and sedimentation. To reduce remaining aldehyde groups, 0.1% (wt/vol) sodium borohydride was added for 10 min. Then, beads were washed three times with PBS and treated with 1% BSA in PBS for 10 min to block non-specific protein binding sites. After blocking, the beads were used for affinity purification of the respective antibody. For this purpose, the Arl3 immune serum diluted 1:1 (v/v) with PBS was added and incubated overnight at 4 °C on a rotating wheel. Next day, the fusion protein beads with the bound antibody were washed several times with PBS. Bound antibodies were eluted with 100 µL of 0.2 M glycine, pH 2.7, for 5 min (on ice). The pH-eluted antibody was neutralized by the addition of 25 µL of 1 M Tris, pH 8.5. The antibody was diluted to a concentration of 0.1 mg/mL and complemented with BSA (0.1 mg/mL final concentration) for stabilization. The Arl3(T31N) antibody was affinity-purified as described above, except for using Arl3(T31N)-MBP for affinity purification.

### 5.2. Immunofluorescence Microscopy of Immunolabeled Semi-Thin Sections/Cryosections by Confocal and Super-Resolution Structured Illumination Microscopy (SR-SIM)

Immunolabeling of cryostat sections was performed as previously described [20,29]. The process of tissue embedding and immunolabeling of 0.5 µm-thin (“semi-thin“) resin sections was performed exactly as described previously [53,65,66,67,76,80,81]. Sections were immunolabeled with the antibody dilutions described above and analyzed on a Nikon A1R confocal microscope, as previously described [53,67,80,81]. Super-resolution structured illumination microscopy (SR-SIM) was performed with an Elyra PS1 setup (Zeiss) equipped with ZEN software, exactly as previously described [53,67,76], using a 63× Plan Apo objective (N.A. 1.4). The FocalCheck™ fluorescence microscope test slide #1 (F36909; Invitrogen, Molecular Probes) coated with FocalCheck™ ring-stained microspheres (green and red) of 1 µm size (No. 4; row A) was used for calibration and alignment correction.

### 5.3. Preparation of Cryostat Section from PFA-Fixed Mouse Retinas

Mice were dark-adapted overnight and sacrificed the next morning in dim red light, as previously described [39]. The mouse eye cornea was punctured with a needle and the lens was removed after cutting the anterior segment in dim red light. The posterior eye cup was immersion-fixed in 4% PFA in PBS, pH 7.4, for 2 h at 4 °C. The eye cup was rinsed with PBS and cryoprotected overnight in 30% sucrose in PBS. The next day, the eye cup was flash frozen in liquid-nitrogen-cooled isopentane and was embedded in NEG50 medium (Richard-Allan Scientific). Then, 12 µm thick sections were prepared on three-fold gelatinized slides and were used for immunolabeling. Sections were washed one time with PBS and blocked with 10% BSA in PBS (blocking buffer). Sections were incubated with Arl13b antibody from NeuroMab (1:200) and RIBEYE antibody U2656 (1:1000) in blocking buffer overnight at 4 °C. Next day sections were washed three times with PBS and incubated with secondary donkey anti-mouse Alexa568 and donkey anti-rabbit Alexa488 for 1 h at RT, and were washed and embedded in N-propyl gallate (NPG) medium [53,82] for visualization. Images were acquired with a Nikon A1R confocal microscope (Nikon, Düsseldorf, Germany) equipped with 488, 561 and 647 nm laser excitation lines and the NIS-Elements software (NIS-Elements AR 3.2, 64 bit, Nikon, Düsseldorf, Germany) using a 60× oil objective (N.A. 1.4).

### 5.4. Triple Immunolabeling Experiments

Triple-immunolabeling analyses with three different primary antibodies (with two antibodies from an identical species; i.e., two rabbit primary antibodies) were performed, as previously described [65,67,82]. In brief, two of the three primary antibodies that were generated in different species (i.e., a mouse primary antibody and a rabbit primary antibody) were incubated simultaneously overnight at 4 °C at the indicated dilutions. After initial incubation with the first primary antibody solutions, cryosections were washed three times with PBS to remove unbound primary antibodies followed by an incubation with the fluorophore-conjugated secondary antibodies (1:1000 dilution; 1 h, RT). Next, sections were blocked using goat polyclonal monovalent anti-rabbit IgG Fab fragments (Rockland Immunochemicals #811-1102 via Biomol (Hamburg, Germany); 1:100 dilution from a 1.0 mg/mL stock; 3 h, RT) depending on the species in which the third primary antibody was generated overnight at 4 °C. After preincubation with Fab fragments and three washes with PBS, the third primary antibody (developed in rabbit) was added and incubated overnight (4 °C) at the indicated dilution. Binding of the third primary antibody was detected with fluorophore-conjugated secondary antibody. Sections were washed with PBS for three times and mounted on glass slides with NPG [53,82]. Controls were performed by omitting one (of the two) primary antibodies generated in the same species to judge on the specificity of the immunosignals and to check for possible cross-talks between the two different immunosignals obtained with the primary antibodies generated in the same species. No cross-talk between channels was observed in these control experiments (bleed-through controls).

### 5.5. PreAbsorption of Antibodies

#### 5.5.1. Arl3

Arl3 serum (WT) was diluted 1:100 in 0.5% BSA in PBS for immunofluorescence microscopy and 1:500 dilution in 3% milk for Western blotting. To one half, 50 μg Arl3-GST was added; to the other half GST alone was added (30 μg). The peptide-antibody mixtures were incubated on a turning wheel overnight (4 °C). On the next day, beads were removed by centrifugation and the supernatants used for WB or IF as indicated.

#### 5.5.2. Arl13b (Abcam)

For preabsorption blocking experiments, the polyclonal Arl13b (Abcam) antibody was diluted 1:500 in PBS. To one half of the antibody dilution, the specific Arl13b-blocking peptide (Antibodies-online, ABIN973421) (25 μg) was added; to the other half, an unrelated control Nphp3 peptide was added (25 μg). The peptide–antibody mixtures were incubated on a turning wheel overnight (4 °C). On the next day, preabsorbed antibodies were used for immunolabeling experiments on mouse retina cryosections.

#### 5.5.3. Nphp3 (Clone 5A3)

Monoclonal Nphp3-N5 (5A3) antibody was diluted 1:500 in 3% milk and blocked with the 20 μg Nphp3-N5 (5A3)-blocking peptide and unrelated control RIM2 peptide [76]. The peptide–antibody mixtures were incubated on a turning wheel overnight (4 °C). The preabsorbed antibody mixture was used for Western blot.

#### 5.5.4. Rp2 (Lab-Made Rabbit Polyclonal Antiserum)

Antiserum was diluted 1:500 in 3% milk. To one volume half of the antibody dilution 50 μg of the Rp2-MBP fusion protein was added; to the other volume half, 25 μg of MBP alone was added. The preabsorbed antibody was used for Western blot.

### 5.6. Immunostaining of PNA-stained Cone Synapses on Cryostat Sections of the Bovine Retina

Visualization of cone synapses with PNA-Alexa568 and immunolabeling of PNA-stained cone terminals was performed exactly as previously described [83]. For the immunolabeling, the rabbit polyclonal Arl13b (Proteintech) was used in a 1:50 dilution in blocking solution, containing 0.5% BSA in PBS. PNA-Alexa568 was diluted 1:200 in blocking buffer, exactly as described [83].

### 5.7. Quantitative Analyses of Immunofluorescence Intensities and Statistical Evaluation

Integrated fluorescence intensities of Nphp3 and actin immunosignals in RIBEYE knockout and control tissue (Figure 9) were measured and quantified with NIH ImageJ (Fiji, version 1.48F), as previously described [53,67,80,81]. Analyses were performed in a blinded manner. A rectangular region of interest (ROI) was drawn in ImageJ around the OPL and the connecting cilium (cc) (identified by the typical actin immunostaining pattern) to determine the fluorescence intensities of each antibody as integrated density. Averages of the intensity values (exported from ImageJ) were calculated and plotted in Microsoft Excel. Values were plotted as relative values (in%) normalized to the control values. Statistical analyses of significance were performed with OriginPro software (OriginLab Corporation, version 2018). Fluorescence intensity data (Figure 9) were not normally distributed as judged by the Shapiro–Wilk test. Therefore, the non-parametric two-tailed Mann–Whitney *U* test (significance level α = 0.05) was used to compare two groups of samples.

### 5.8. Pre-Embedding Immunogold Labeling (Rp2/Arl3(T31N))

Pre-embedding immunogold electron microscopy was performed as previously described [84] with some modifications. First, 20 μm-thick retina cryostat sections were collected on gelatin-coated glass cover slides and briefly fixed for 2 min with 2% freshly depolymerized PFA in PBS. For pre-embedding immunogold analyses with Rp2 antibodies, cryostat sections from flash-frozen isolated bovine retinas were used. For Arl3(T31N), cryostat sections from flash-frozen mouse eyes were used. After fixation, sections were washed with PBS (5 × 10 min each). Next, sections were treated with 0.5% BSA in PBS (blocking buffer) for 1 h (RT) to block unspecific binding sites and incubated with the indicated primary antibody dilutions (24 h, 4 °C) (Rp2/Arl3(T31N) diluted 1:100 in blocking buffer). No primary antibody served as a negative control. After several washes with PBS to remove the unbound primary antibody, sections were incubated with goat anti-rabbit secondary antibody conjugated to ultrasmall (1.4 nm diameter) gold particles (1:50 dilution in blocking buffer; 24 h, 4 °C). Unbound secondary antibody was removed by several washes with PBS, and sections were then fixed with 2% glutaraldehyde (10 min RT) and washed with water 5 × 10 min each. Then, ultrasmall gold particles were silver enhanced for 20 min in the dark using a commercial silver enhancement kit (HQ Silver Enhancement Kit; Nanoprobes). After silver enhancement, the sections were processed exactly as previously described [84].

### 5.9. Post-Embedding Immunogold Labeling (RIBEYE/Nphp3/Arl13b)

Post-embedding immunogold labeling was performed largely as previously described [29,65]. Ultrathin sections from bovine/mouse retina were incubated with 0.5 % bovine serum albumin (BSA) in PBS (1 h, RT) to block non-specific protein binding sites. Then, sections were incubated with primary antibodies, i.e., anti-RIBEYE antibody U2656 (1:5000 dilution in 0.5% BSA/PBS, denoted as blocking buffer), anti-RIBEYE antibody 2D9 (1:100 dilution in blocking buffer), Nphp3-N5 (clone 5A3; 1:200 dilution in blocking buffer) and anti-Arl13b (Abcam, 1:100 in blocking buffer) overnight at 4 °C. Binding of mouse monoclonal RIBEYE primary antibody (clone 2D9) was detected with goat anti-mouse secondary antibody conjugated to 5 nm gold particles (1:100 dilution in blocking solution); binding of RIBEYE rabbit polyclonal antibody (U2656) and Arl13b rabbit polyclonal antibody (Abcam) was detected by goat anti-rabbit secondary antibody conjugated to 5 nm gold particles (1:100 dilution in blocking solution) for 1 h at RT. For Nphp3 (clone 5A3), sections were treated with rabbit anti-mouse IgA bridging antibody (diluted 1:100 in blocking solution) for 2 h at RT. After several washes with PBS, binding of the bridging antibody was detected with goat anti-rabbit secondary antibody conjugated to 5 nm gold particles (1:100 dilution in blocking solution) for 1 h at RT. After several washes with PBS, immune complexes were fixed with 2.5% glutaraldehyde in PBS for 15 min at RT. Then, sections were washed with water and contrasted with 2% uranylacetate in H_2_O for 15 min at RT. Finally, after several washes with water, sections were air dried. As negative controls, either primary antibodies were omitted and/or unrelated antibodies were used. Samples were analyzed with Tecnai Biotwin 12 digital microscopy (FEI) and iTEM software.

### 5.10. Quantification of Post-Embedding Immunogold Labeling Data

Electron micrographs of immunolabeled rod photoreceptor synapses were obtained at a magnification of 43,000×. The areas of the rod presynaptic terminals and of the synaptic ribbon were measured with ImageJ (Fiji, version 1.48F) using the free-hand tool. The scale bar of the EM images was used for normalization. In these micrographs, the immunogold particles at the synaptic ribbon and in the entire presynaptic terminal were counted manually. For the analysis, the immunogold particle density at the synaptic ribbon in comparison to the immunogold particle density in the remaining presynaptic terminal were calculated with Microsoft Excel. Raw immunogold density values were exported to OriginPro software (version 69.4.0.220, OriginLab Corporation) for statistical analyses. The Shapiro–Wilk test was used for the analyses of normal distribution. Data were not normally distributed. For the analyses of the dependent values (paired samples), the non-parametric Wilcoxon signed-rank test was used to calculate statistical significance. For the analysis of immunogold particle density along the synaptic ribbon, the synaptic ribbon was subdivided into three parts, i.e., the proximal 100 nm anchored to the active zone and which includes the proper active zone, the middle 100 nm and the remaining distal and membrane-distal portion using the straight line tool in ImageJ (Fiji version 1.48F).

### 5.11. Multipeptide Arrays: Dot Blot Assays

For antibody epitope mapping, peptides of Nphp3 covering the N-terminus (aa 1–127) with a length of 15 amino acids each and an overlap of 13 amino acids were synthesized on a hardened cellulose membrane with a ResPep SL Synthesizer (Intavis Bioanalytical Instruments; Cologne, Germany) as described [85]. Membranes were activated in methanol for 4 min and then equilibrated in binding buffer (0.1% Triton X-100 in PBS) for 2 h. After incubation in blocking buffer (1 µM BSA in binding buffer, 1 h, RT), the membrane was incubated overnight (at 4 °C) in primary antibody solution, i.e., Nphp3-N4 diluted 1:2000 in blocking buffer or Nphp3-N1 diluted 1:1000 in blocking buffer. After washing three times (5 min each, RT), the membrane was incubated with the secondary antibody goat anti-rabbit coupled to peroxidase (1:3000 dilution in blocking buffer, 1 h, RT). The bound Nphp3-antibodies were visualized by enhanced chemiluminescence using a Bio-Rad GelDoc apparatus.

### 5.12. Miscellaneous Methods

SDS-PAGE and Western blotting experiments were performed as previously described [29].

## Figures and Tables

**Table 1:** Additional primary antibodies used in the study.

Primary Antibodies	References/Source	Dilution
Anti-RIBEYE mouse monoclonal antibody (2D9) against the C-terminus of RIBEYE(B)-domain/CtBP2	[67]	1:200 (IF) (≈2.5 µg/mL final concentration); 1:100 (WB) (≈5 µg/mL final concentration)
Anti-RIM1/2 rabbit polyclonal antibody	[74,75,76]	1:200 (IF)
Anti-SV2 mouse monoclonal antibody	[77]	1:50 (IF)
Anti-GFAP rabbit polyclonal IgG	Sigma (Taufkirchen, Germany), G9269	1:300 (WB)
Anti-actin (clone C4) mouse monoclonal antibody	Millipore (Darmstadt, Germany), MAB1501	1:100 (IF) 1:2000 (WB)
Anti-RIBEYE(B)/CtBP2 mouse monoclonal antibody	BD Transduction Laboratories (Heidelberg, Germany), 612044	1:1000 (IF)
DyLight 650 directly labeled anti-RIBEYE(B)/CtBP2	[65]	1:20 (IF)
Anti-PSD95 rabbit polyclonal (L667)	[78]	1:750 (IF)
Anti-RIBEYE rabbit polyclonal antibody (U2656) against RIBEYE(B)-domain	[29]	1:1000 (IF) 1:5000 (EM)

**Table 2:** Secondary antibodies used in the study.

Antibody	Source	Dilution
Chicken anti-mouse Alexa488	Invitrogen Molecular Probes (Karlsruhe, Germany), A-21200	1:1000 (IF)
Donkey anti-mouse Alexa488	Invitrogen Molecular Probes (Karlsruhe, Germany), A-21202	1:1000 (IF)
Donkey anti-rabbit Alexa568	Invitrogen, Molecular Probes (Karlsruhe, Germany), A-10042	1:1000 (IF)
Chicken anti-rabbit Alexa488	Invitrogen, Molecular Probes (Karslruhe, Germany), A-21441	1:1000 (IF)
Donkey anti-mouse Alexa568	Invitrogen, Molecular Probes (Karlsruhe, Germany), A-10037	1:1000 (IF)
Goat anti-mouse Alexa647	Invitrogen, Molecular Probes (Karlsruhe, Germany), A-21236	1:1000 (IF)
Donkey anti-mouse Alexa647	Invitrogen, Molecular Probes (Karslruhe, Germany), A-31571	1:1000 (IF)
Goat anti-rabbit peroxidase-conjugated (POX) IgG	Sigma (Taufkirchen, Germany), A-6154	1:3000 (WB)
Goat anti-mouse (IgG, IgM, IgA), conjugated to peroxidase	Sigma (Taufkirchen, Germany), SAB3701048	1:3000 (WB)
Goat anti-mouse POX IgG	Sigma (Taufkirchen, Germany), A-3673	1:3000 (WB)
Monovalent Fab fragments goat anti-rabbit (unconjugated) (H&L)	Rockland Immunochemicals, 811–1102 via Biomol GmbH	1:100 (IF)
Rabbit anti-mouse IgA	Novus (Wiesbaden, Germany), NB7506	1:100 (IF) 1:1000(WB) 1:100 (EM)
Goat anti-rabbit antibody conjugated to ultrasmall (1.4 nm-diameter) gold particles	Nanoprobes, #2003 via Biozol (Eching, Germany)	1:50 (EM)
Goat anti-rabbit secondary antibody conjugated to 5 nm gold particles	Sigma (Taufkirchen, Germany), G7277	1:100 (EM)
Goat anti-mouse secondary antibody conjugated to 5 nm gold particles	Sigma (Taufkirchen, Germany), G7527	1:100 (EM)

## Data Availability

All data are presented in the main manuscript and the Supplementary Materials.

## Supplementary Materials

The following supporting information can be downloaded at: https://www.mdpi.com/article/10.3390/ijms23137135/s1.
