# Supplementary material for: Ciliary Proteins Repurposed by the Synaptic Ribbon: Trafficking Myristoylated Proteins at Rod Photoreceptor Synapses
Source: Int J Mol Sci. 2022 Jun 27;23(13):7135. doi: 10.3390/ijms23137135 (PMC9266639; doi:10.3390/ijms23137135)
Supplement: Supplementary file 1 [file ijms-23-07135-s001.zip › ijms-1754517-supplementary.pdf]

**Supplementary Figures**

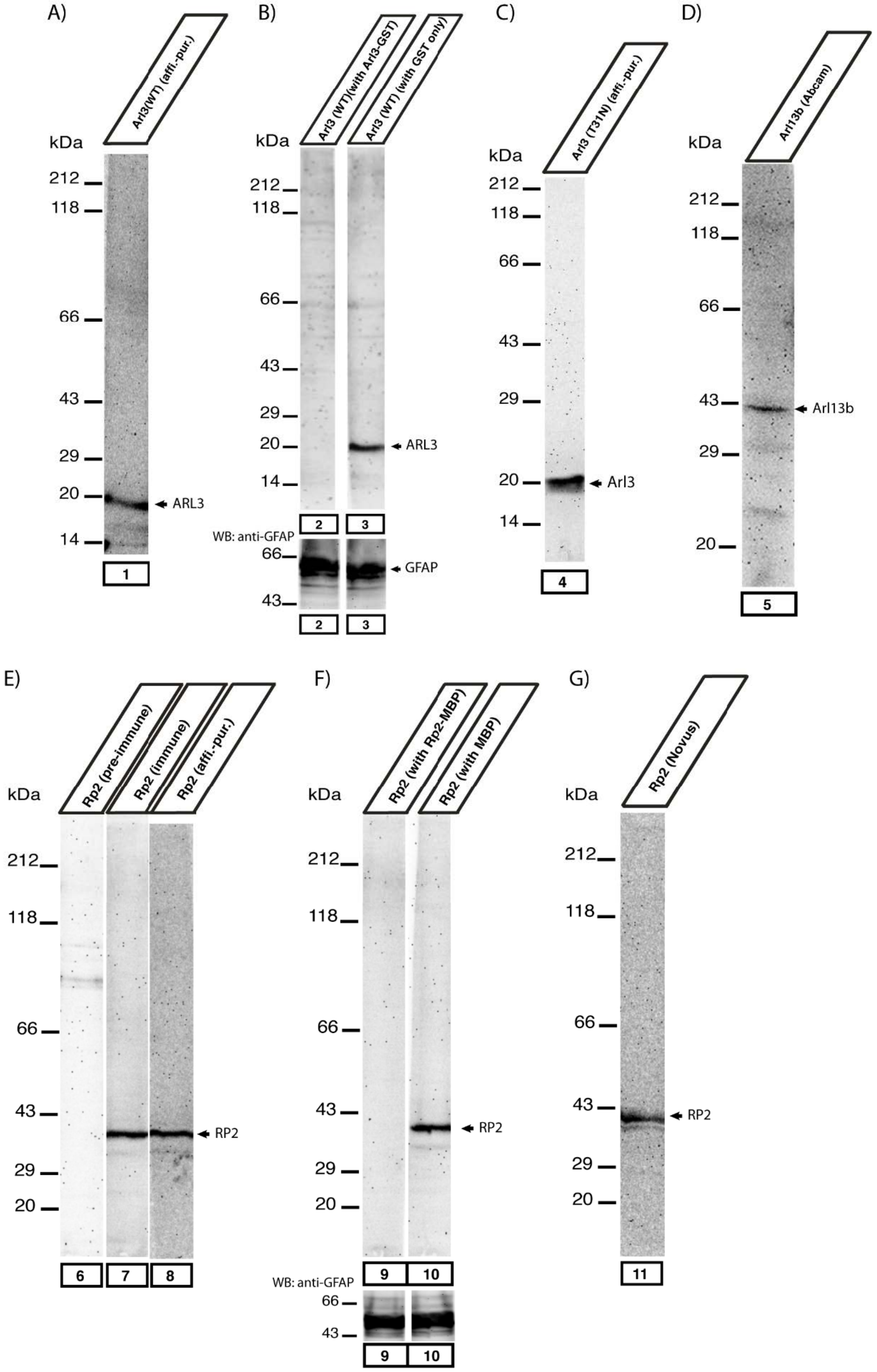

**Supplemental Figure S1. Retina lysates probed with the indicated antibodies by Western blot.** Arl3 antibodies detected a single band at ≈20 kDa (A, lane 1) in bovine retina lysate that was absent if the antibody was preabsorbed with Arl3-GST (B, lane 2, upper panel) but not after preabsorption with GST only (B, lane 3, upper panel). Incubation of the same strips with antibodies against GFAP served as loading controls (B, lanes 2, 3, lower panel). Arl3(T31N) affinity-purified antibodies detected an Arl3-typical WB band (C, lane 4) in mouse retina lysate. Antibodies against Arl13b (Abcam) detected a major band at ≈40 kDa (D, lane 5) in mouse retina lysate. Rp2 immune serum (E, lane 7) as well as affinity-purified Rp2 antibody (E, lane 8), but not Rp2 preimmune serum (E, lane 6), detected a single protein band at the expected running position of ≈40 kDa in bovine retina lysate. This band could be blocked by preabsorption of the antibody with Rp2-MBP fusion protein (F, lane 9, upper panel) but not by preabsorption with MBP alone (F, lane 10, upper panel). Incubation of the same strips with antibodies against GFAP served as loading controls (F, lanes 9-10, lower panel). Another commercially available antibody against Rp2 used in this study also detected a single band at ≈40 kDa (G, lane 11) in bovine retina lysate. Abbreviations: GFAP, glial fibrillary acidic protein.

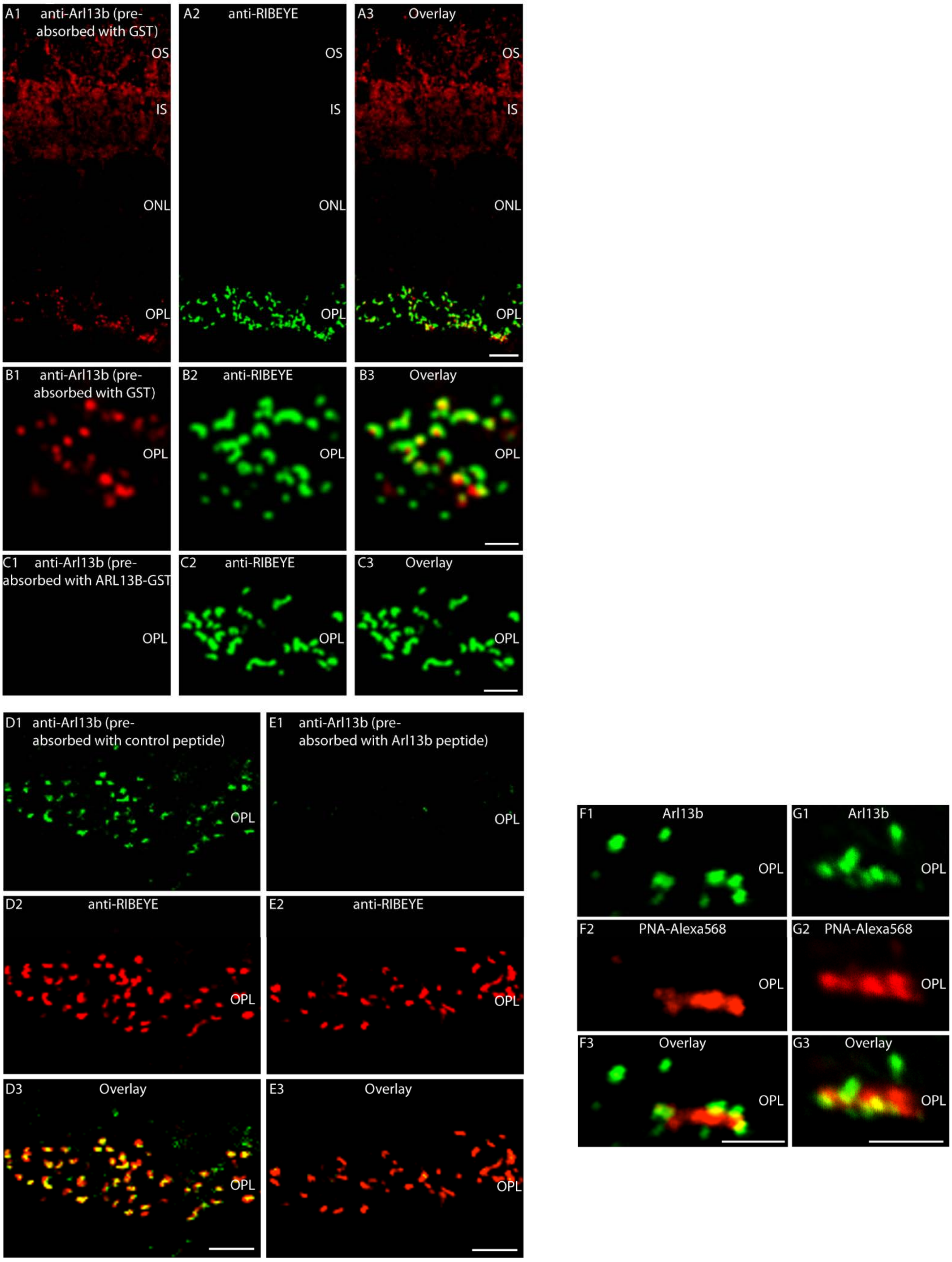


**Supplemental Figure S2**. **Preabsorption of Arl13b antibodies (Proteintech/Abcam) (A-C).** (**A1-A3, B1-B3, C1-C3**) Cryostat sections of the bovine retina immunolabeled with mouse monoclonal antibodies against RIBEYE (clone 2D9) (A2, B2, C2) and affinity-purified rabbit polyclonal antibodies against Arl13b (Proteintech) (A1, B1, C1) that were preabsorbed as indicated below. In addition to immunolabeling of the outer and inner segments, the affinity-purified Arl13b antibody produced a punctated immunosignal in the OPL at the synaptic ribbons that were labeled with RIBEYE antibodies (A, B). Preabsorption of the Arl13b antibodies with Arl13b-GST (C1), but not with GST alone (A1, B1), abolished synaptic Arl13b immunosignals in the OPL at the synaptic ribbon whereas RIBEYE immunosignals (A2, B2, C2) were unaffected by both treatments. Immunosignals of the two channels

were overlaid in (A3, B3, C3). (**D-E**) Cryostat sections of the mouse retina were double-immunolabeled with mouse anti-RIBEYE antibody (clone 2D9) and rabbit Arl13b antibody (Abcam) that was preabsorbed either with the peptide used for immunization (D2) or with an unrelated Nphp3 peptide (E2). Preabsorption with the Arl13b peptide abolishes Arl13b immunostaining of the OPL whereas preabsorption with Nphp3 control peptide has no effect on the Arl13b immunosignals in the OPL. Immunosignals of the two channels were overlaid in (D3, E3). (**F-G**) Cryostat sections of the bovine retina were double-labeled with rabbit Arl13b antibody (Proteintech) (F1, G1) and PNA-Alexa568 (F2, G2) to label cone terminals in the OPL. Signals of the two channels were overlaid in (F3, G3). Abbreviations: OS, outer segment; IS, inner segment; ONL, outer nuclear layer; OPL, outer plexiform layer. Scale bars: 5 µm (A, D, E), 3 µm (B, C, F, G).

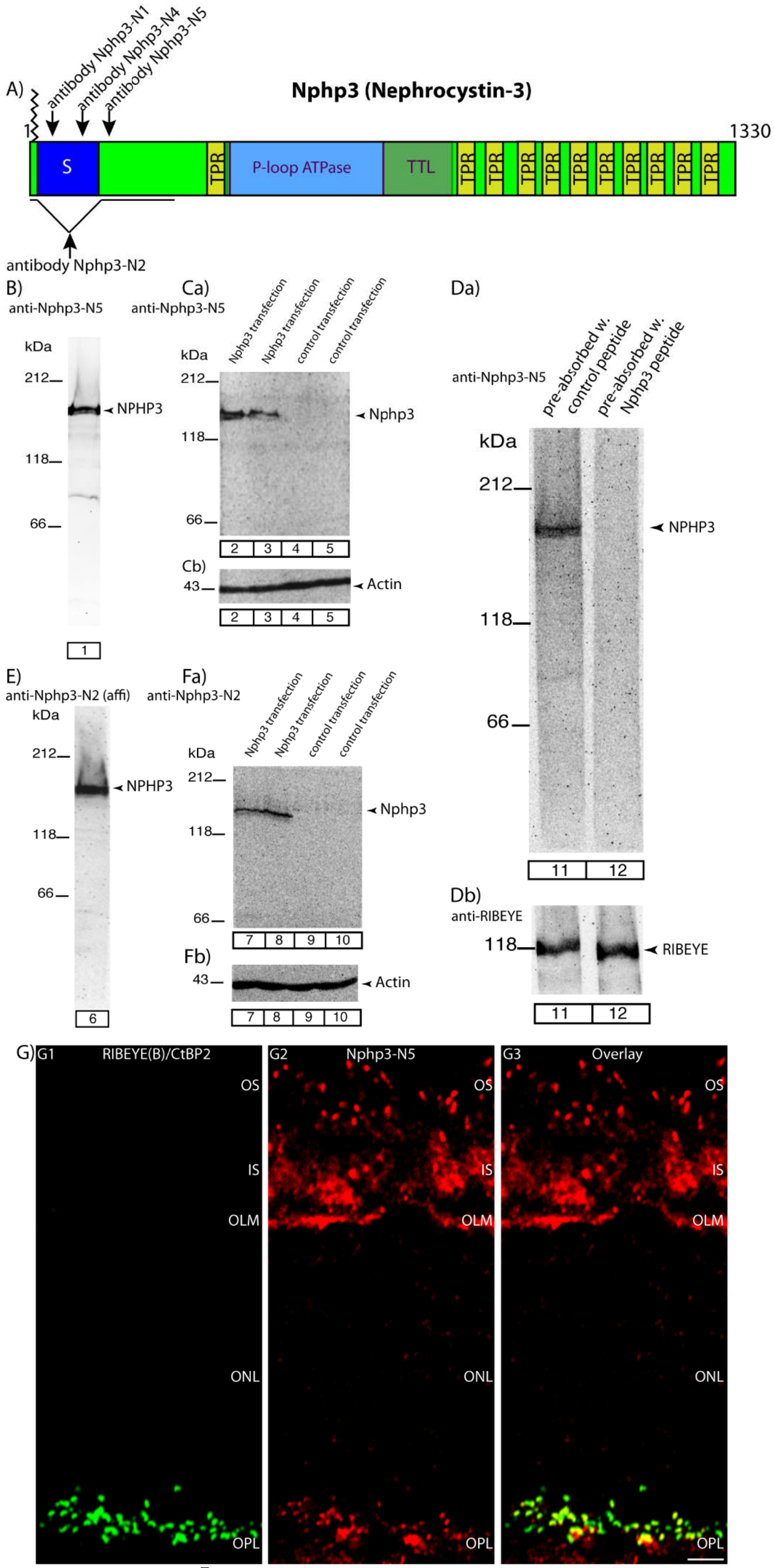
A)
antibody Nphp3-N1
antibody Nphp3-N4
antibody Nphp3-N5
Nphp3 (Nephrocystin-3)
1
1330
S
TPR
P-loop ATPase
TTL
antibody Nphp3-N2
B)
anti-Nphp3-N5
kDa
212
118
66
NPHP3
1
Ca)
anti-Nphp3-N5
Nphp3 transfection
Nphp3 transfection
control transfection
control transfection
kDa
212
118
66
Nphp3
2 3 4 5
Cb)
43
Actin
2 3 4 5
Da)
anti-Nphp3-N5
pre-absorbed w. control peptide
pre-absorbed w. Nphp3 peptide
kDa
212
118
66
NPHP3
11 12
Db)
anti-RIBEYE
118
RIBEYE
11 12
E)
anti-Nphp3-N2 (affi)
kDa
212
118
66
NPHP3
6
Fa)
anti-Nphp3-N2
Nphp3 transfection
Nphp3 transfection
control transfection
control transfection
kDa
212
118
66
Nphp3
7 8 9 10
Fb)
43
Actin
7 8 9 10
G)
G1 RIBEYE(B)/CtBP2
G2 Nphp3-N5
G3 Overlay
OS
IS
OLM
ONL
OPL

**Supplemental Figure S3**. **Characterization of Nphp3 antibodies.** (**A**) Schematic depiction of Nphp3 (nephrocystin-3) domain structure. Sequence stretches representing the antigens to which antibodies Nphp3-N1, Nphp3-N2, Nphp3-N4 and Nphp3-N5 were raised are indicated (arrows). Abbreviations: S, N-terminal alternatively spliced exon; TPR, tetratricopeptide repeat; TTL, tubulin-tyrosine ligase domain. Nphp3 is myristoylated at the N-terminus (aa 2). (**B-F**) Western blot characterization of the anti-Nphp3 antibodies Nphp3-N2 and Nphp3-N5. (B, E), Western blot analyses of Nphp3-N5 (B) and Nphp3-N2 (E) antibodies tested with bovine retina lysate. Nphp3-N5 (C) and Nphp3-N2 (F) were tested by using HEK293 cells that were either transfected with Nphp3 cDNA or control cDNA. Only in Nphp3-transfected HEK cells a single band at the expected running position for Nphp3 was observed (upper panels in C and F). In the lower panels of (C) and (F), the nitrocellulose membranes as shown in the upper panels were re-probed with anti-actin antibodies (as loading control). (D) Nphp3-N5 antibody was preabsorbed either with the Nphp3 peptide used for immunization (D, lane 12) or with control peptide (D, lane 11) and tested in Western blot analyses on bovine retina lysate. Only in the control (preabsorbed with control peptide) was the typical Nphp3 Western blot band observed (lane 11, upper panel); this band was completely absent if the antibody was preabsorbed with the Nphp3 peptide (lane 12, upper panel). In the lower panel, the same nitrocellulose membrane as shown in the upper panel, was re-probed with antibodies against RIBEYE (as loading control). (**G1-G3**) Cryostat section of the bovine retina immunolabeled with anti-Nphp3-N5 antibody (clone 5A3). Synaptic ribbons were visualized by fluorescently labeled primary mouse monoclonal antibody against RIBEYE(B)/CtBP2 (directly labeled primary antibody). In addition to immunolabeling of the outer and inner segments, the Nphp3-N5 mouse monoclonal antibody produced punctated immunosignals in the OPL, where the photoreceptor synapses are located, close to the synaptic ribbons (for high-resolution analyses of Nphp3 localization, see Figs. 7, 8). (G) was obtained by confocal microscopy. Abbreviations: OS, outer segment; IS, inner segment; OLM, outer limiting membrane; ONL, outer nuclear layer; OPL, outer plexiform layer. Scale bar: 5 μm (G).

**Nphp3-N1**

**A** 1 70
MGTASSLVSPAGGEVIEDTYGAGGGEACEIPVEVKPKARLLRNSFRRGAGAAAGAGPGSLPRGVGAGGLL
GASFKSTGSSVPELEYAAAEYERLRKEYEIFRVSKNQELLSMGRREAKLDTENKRLRAELQ 131

**B**

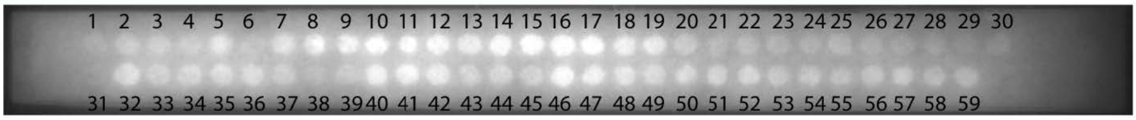


**C**

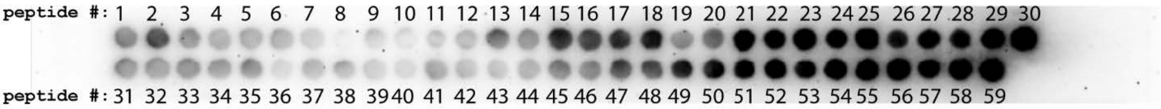


**D**

aa #:25 GEACEIPVEVKPKARLLRNSFRRGAGAAAGAGPGSLPRGVGAGGLLGAS 73
peptide #:13 GEACEIPVEVKPKAR
peptide #:14 ACEIPVEVKPKARLL
peptide #:15 EIPVEVKPKARLLRN
peptide #:16 PVEVKPKARLLRNSF
peptide #:17 EVKPKARLLRNSFRR
peptide #:18 KPKARLLRNSFRRGA
peptide #:19 KARLLRNSFRRGAGA
peptide #:20 RLLRNSFRRGAGAAA
peptide #:21 LRNSFRRGAGAAAGA
peptide #:22 NSFRRGAGAAAGAGP
peptide #:23 FRRGAGAAAGAGPGS
peptide #:24 RGAGAAAGAGPGSLP
peptide #:25 AGAAAGAGPGSLPRG
peptide #:26 AAAGAGPGSLPRGVG
peptide #:27 AGAGPGSLPRGVGAG
peptide #:28 AGPGSLPRGVGAGGL
peptide #:29 PGSLPRGVGAGGLLG
peptide #:30 SLPRGVGAGGLLGAS

aa #:93 RLRKEYEIFRVSKNQELLSMGRREAKLDTENKRLRAELQ 131
peptide #:47 RLRKEYEIFRVSKNQ
peptide #:48 RKEYEIFRVSKNQEL
peptide #:49 EYEIFRVSKNQELLS
peptide #: 50 EIFRVSKNQELLSMG
peptide #: 51 FRVSKNQELLSMGRR
peptide #: 52 VSKNQELLSMGRREA
peptide #: 53 KNQELLSMGRREAKL
peptide #:54 QELLSMGRREAKLDT
peptide #: 55 LLSMGRREAKLDTEN
peptide #: 56 SMGRREAKLDTENKR
peptide #: 57 GRREAKLDTENKRLR
peptide #: 58 REAKLDTENKRLRAE
peptide #: 59 AKLDTENKRLRAELQ

**Nphp3-N4**

**E** 1 70
MGTASSLVSPAGGEVIEDTYGAGGGEACEIPVEVKPKARLLRNSFRRGAGAAAGAGPGSLPRGVGAGGLL
GASFKSTGSSVPELEYAAAEYERLRKEYEIFRVSKNQELLSMGRREAKLDTENKRLRAELQ 131

**F**

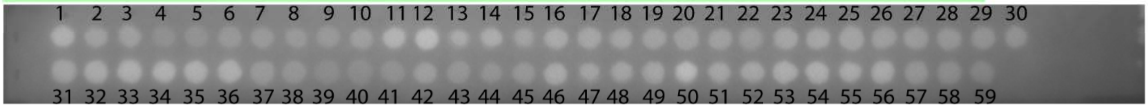


**G**

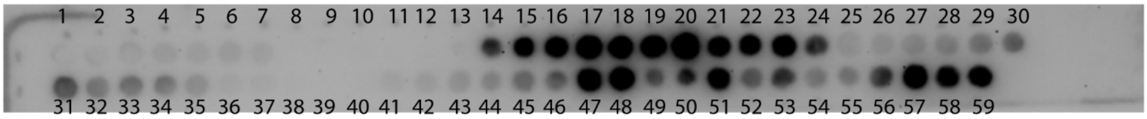


**H**

aa #:27 ACEIPVEVKPKARLLRNSFRRGAGAAAGAGPGSLP 61
peptide #:14 ACEIPVEVKPKARLL
peptide #: 15 EIPVEVKPKARLLRN
peptide #: 16 PVEVKPKARLLRNSF
peptide #: 17 EVKPKARLLRNSFRR
peptide #: 18 KPKARLLRNSFRRGA
peptide #: 19 KARLLRNSFRRGAGA
peptide #: 20 RLLRNSFRRGAGAAA
peptide #: 21 LRNSFRRGAGAAAGA
peptide #: 22 NSFRRGAGAAAGAGP
peptide #: 23 FRRGAGAAAGAGPGS
peptide #: 24 RGAGAAAGAGPGSLP

aa #:93 RLRKEYEIFRVSKNQELLSMGRREAKLDTENSMGRREAKLDTENKRLRAELQ 131
peptide #:47 RLRKEYEIFRVSKNQ
peptide #: 48 RKEYEIFRVSKNQEL
peptide #: 49 EYEIFRVSKNQELLS
peptide #: 50 EIFRVSKNQELLSMG
peptide #: 51 FRVSKNQELLSMGRR
peptide #: 52 VSKNQELLSMGRREA
peptide #: 53 KNQELLSMGRREAKL

peptide #: 56 SMGRREAKLDTENKR
peptide #: 57 GRREAKLDTENKRLR
peptide #: 58 REAKLDTENKRLRAE
peptide #: 59 AKLDTENKRLRAELQ

**Supplemental Figure S4. Characterization of Nphp3 antibodies.** The overlapping 12mer peptides derived from the alternatively spliced N-terminal exon highlighted in green (see also Figure 7 A; Supplementary Figure 3 A), were spotted on cellulose acetate membrane and tested for immunoreactivity with the antibodies anti-Nphp3-N1 (A - D) and Nphp3-N4 (E - H). The sequence of the probed immunoreactive peptides is given in (D) and (H). The sequence stretch with the strongest immunoreactivity with the respective antibodies is highlighted in red in panels (A) and (E).

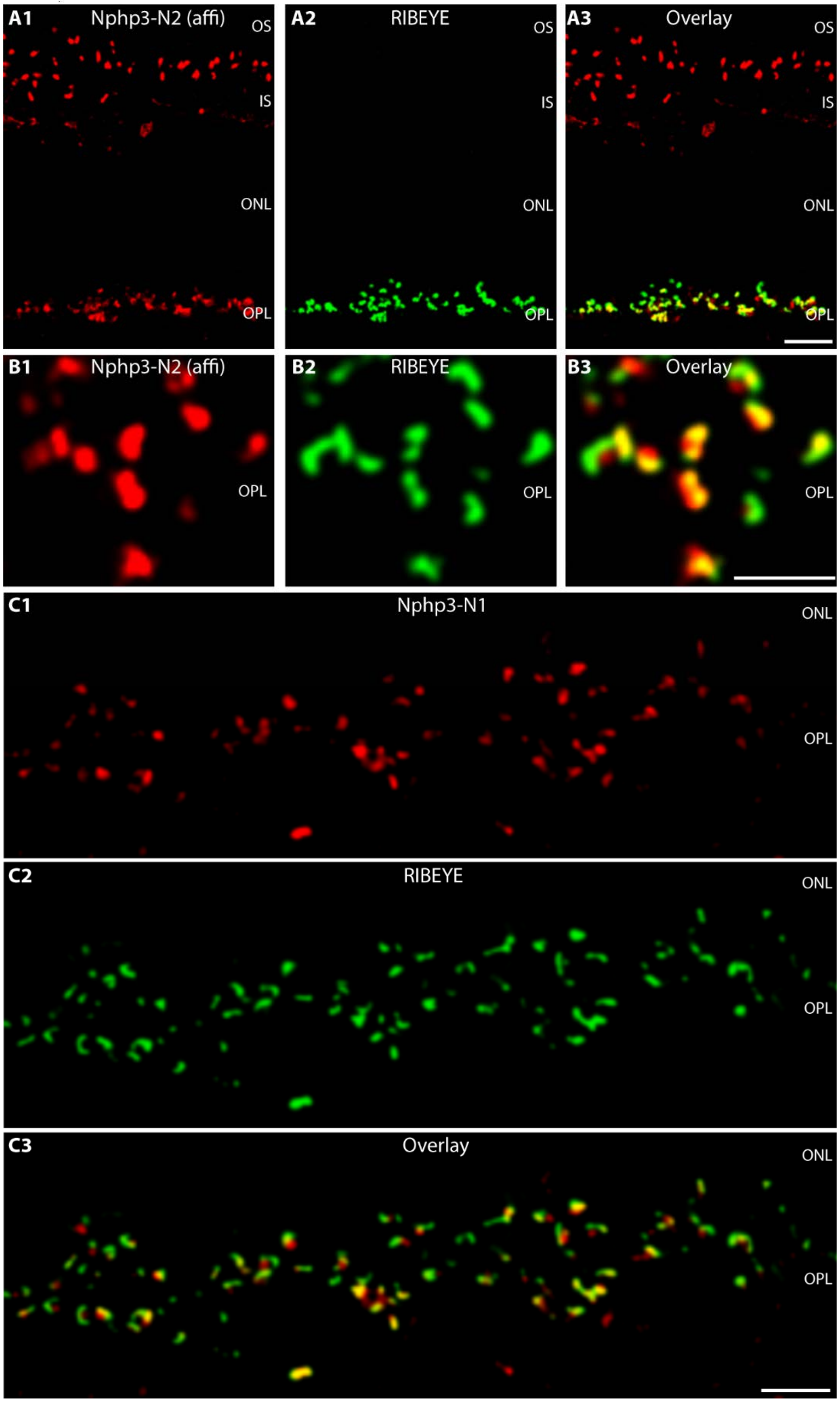


**Supplemental Figure S5. Immunolocalization of Nphp3 in the retina: immunolabeling with different Nphp3 antibodies.** (**A-C**) Cryostat sections of the bovine retina (A, B) and mouse retina (C) immunolabeled with rabbit polyclonal antibody Nphp3-N2 (A1, B1) / Nphp3-N1 (C1) and mouse monoclonal antibody against RIBEYE(B) (clone 2D9) (A2, B2, C2). Images were obtained by confocal microscopy. Abbreviations: OS, outer segment; IS, inner segment; ONL, outer nuclear layer; OPL, outer plexiform layer. Scale bars: 5 µm (A-C).

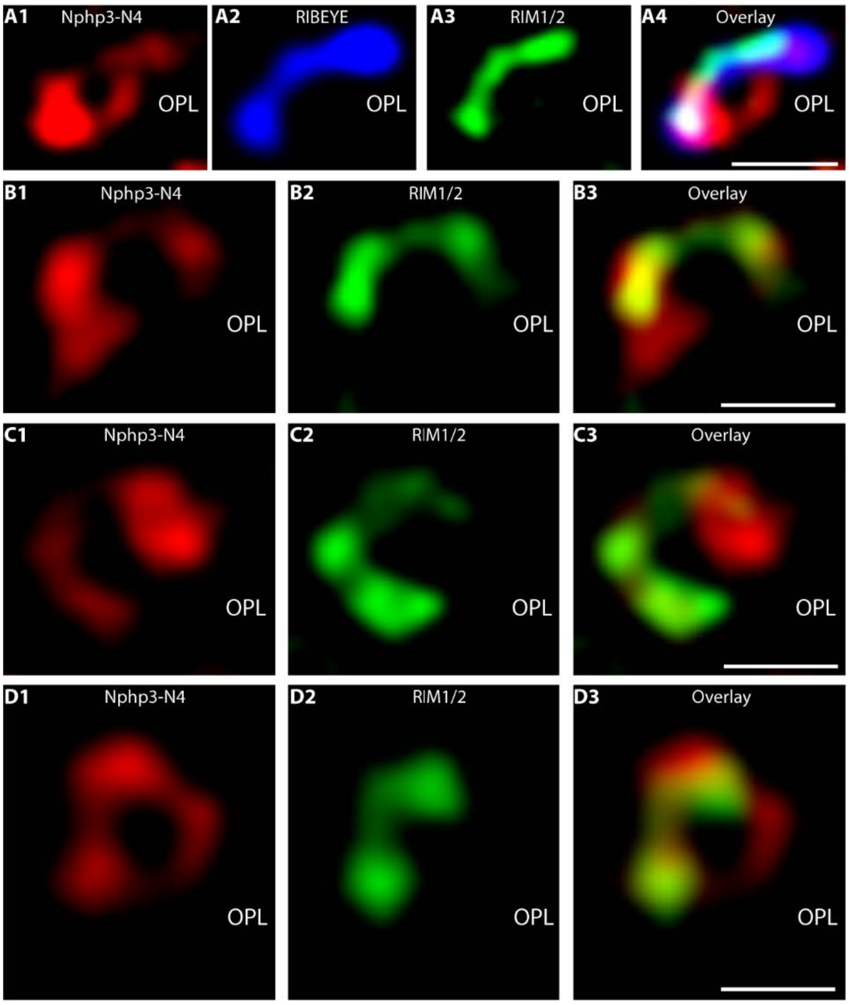


**Supplemental Figure S6**. **High-resolution light microscopical immunolocalization of Nphp3 (SR-SIM) at the synaptic ribbon.** (**A1-A4, B1-B3, C1-C3, D1-D3**) Cryostat sections of the bovine retina immunolabeled with rabbit polyclonal antibody against Nphp3-N4, rabbit polyclonal antibody against RIM1/2 [74–76] and mouse monoclonal antibody against RIBEYE(B)/CtBP2 (BD Transduction Labs**)**, as indicated in the figure. Images were obtained by SR-SIM. Abbreviations: OPL, outer plexiform layer. Scale bars: 1 μm (A - D).
